# Coding and small non-coding transcriptional landscape of tuberous sclerosis complex cortical tubers: implications for pathophysiology and treatment

**DOI:** 10.1038/s41598-017-06145-8

**Published:** 2017-08-14

**Authors:** James D. Mills, Anand M. Iyer, Jackelien van Scheppingen, Anika Bongaarts, Jasper J. Anink, Bart Janssen, Till S. Zimmer, Wim G. Spliet, Peter C. van Rijen, Floor E. Jansen, Martha Feucht, Johannes A. Hainfellner, Pavel Krsek, Josef Zamecnik, Katarzyna Kotulska, Sergiusz Jozwiak, Anna Jansen, Lieven Lagae, Paolo Curatolo, David J. Kwiatkowski, R. Jeroen Pasterkamp, Ketharini Senthilkumar, Lars von Oerthel, Marco F. Hoekman, Jan A. Gorter, Peter B. Crino, Angelika Mühlebner, Brendon P. Scicluna, Eleonora Aronica

**Affiliations:** 1 Department of (Neuro) Pathology, Academic Medical Center, University of Amsterdam, Amsterdam, The Netherlands; 2 GenomeScan BV, Leiden, The Netherlands; 3 Department of Pathology, University Medical Center Utrecht, Utrecht, The Netherlands; 4 Department of Neurosurgery, Rudolf Magnus Institute for Neuroscience, University Medical Center, Utrecht, The Netherlands; 5 Department of Pediatric Neurology, Rudolf Magnus Institute for Neuroscience, University Medical Center, Utrecht, The Netherlands; 6 Department of Pediatrics, Medical University Vienna, Vienna, Austria; 7 Institute of Neurology, Medical University Vienna, Vienna, Austria; 8 Department of Pediatric Neurology, 2nd Faculty of Medicine and Motol University Hospital, Prague, Czech Republic; 9 Department of Pathology and Molecular Medicine, 2nd Faculty of Medicine and Motol University Hospital, Prague, Czech Republic; 10 Department of Neurology and Epileptology, The Children’s Memorial Health Institute, and Department of Child Neurology, Warsaw Medical University, Warsaw, Poland; 11 Pediatric Neurology Unit - UZ Brussel, Brussels, Belgium; 12 Department of Development and Regeneration-Section Pediatric Neurology, University Hospitals KU Leuven, Leuven, Belgium; 13 Systems Medicine Department, Child Neurology and Psychiatry Unit, Tor Vergata University Hospital of Rome, Rome, Italy; 14 Department of Medicine, Brigham and Women’s Hospital, Boston, Massachusetts USA; 15 Department of Translational Neuroscience, Brain Center Rudolf Magnus, University Medical Center Utrecht, Utrecht, The Netherlands; 16 Swammerdam Institute for Life Sciences, Center for Neuroscience, University of Amsterdam, Amsterdam, The Netherlands; 17 Department of Neurology, University of Maryland School of Medicine, Baltimore, MD USA; 18 Center for Experimental & Molecular Medicine, and Department of Clinical Epidemiology, Biostatistics and Bioinformatics, Academic Medical Center University of Amsterdam, Amsterdam, The Netherlands; 19 Stichting Epilepsie Instellingen Nederland (SEIN), Heemstede, The Netherlands

**Keywords:** Computational biology and bioinformatics, Genetics research, Epilepsy

## Abstract

Tuberous Sclerosis Complex (TSC) is a rare genetic disorder that results from a mutation in the *TSC1* or *TSC2* genes leading to constitutive activation of the mechanistic target of rapamycin complex 1 (mTORC1). TSC is associated with autism, intellectual disability and severe epilepsy. Cortical tubers are believed to represent the neuropathological substrates of these disabling manifestations in TSC. In the presented study we used high-throughput RNA sequencing in combination with systems-based computational approaches to investigate the complexity of the TSC molecular network. Overall we detected 438 differentially expressed genes and 991 differentially expressed small non-coding RNAs in cortical tubers compared to autopsy control brain tissue. We observed increased expression of genes associated with inflammatory, innate and adaptive immune responses. In contrast, we observed a down-regulation of genes associated with neurogenesis and glutamate receptor signaling. MicroRNAs represented the largest class of over-expressed small non-coding RNA species in tubers. In particular, our analysis revealed that the miR-34 family (including miR-34a, miR-34b and miR-34c) was significantly over-expressed. Functional studies demonstrated the ability of miR-34b to modulate neurite outgrowth in mouse primary hippocampal neuronal cultures. This study provides new insights into the TSC transcriptomic network along with the identification of potential new treatment targets.

## Introduction

Tuberous Sclerosis Complex (TSC) is a genetic disorder caused by mutations in either *TSC1* or *TSC2* genes, leading to the development of benign lesions/hamartomas in multiple organs^1, 2^. TSC often compromises the central nervous system resulting in complex neurological manifestations consisting of varying combinations of neurodevelopmental delay (including autism), various psychiatric disorders and severe epilepsy^3–5^. Cortical tubers, a form of focal cortical dysplasia, are thought to be major contributors to these disabling neurological manifestations in TSC and are targeted for surgical resection in TSC patients with pharmacologically intractable epilepsy^6–8^. Although the current surgical and pharmacological management of seizures in TSC often provide significant benefits^5, 7, 9, 10^, there is further need for a better understanding of the molecular and physiological basis of the neurological manifestations seen in TSC^11^.

Previous gene expression studies on TSC tuber specimens focused either on selected cDNA sequences^12, 13^ or used mRNA^14^ or microRNA (miRNA) microarray hybridization platforms^15^. Advances in high-throughput RNA sequencing (RNA-seq) technology coupled with sophisticated bioinformatics methods have provided a revolutionary means to systematically map transcriptional units of the human genome^16, 17^. Indeed, RNA-seq has led to a more profound appreciation of the intricate nature of both the coding and non-coding transcriptome of the human brain^18, 19^. Moreover, the non-coding units of the human transcriptome, particularly small RNA species that include miRNA, have emerged as important modifiers of the protein coding transcriptome and, in turn, disease phenotype^19–22^. A comprehensive, parallel scan of both the protein coding and small non-coding brain transcriptome of TSC patients by RNA-seq has never been performed. Thus, we here aimed to first map the protein coding and small non-coding RNA species in the brain transcriptome of TSC patients and second, to identify significantly altered cellular signaling pathways in TSC patients that may be modified by miRNA for a better delineation of the complex pathological signaling pathways and networks seen in TSC.

## Results

### The protein coding transcriptome of tuberous sclerosis complex brain tissue

**Figure 1:**
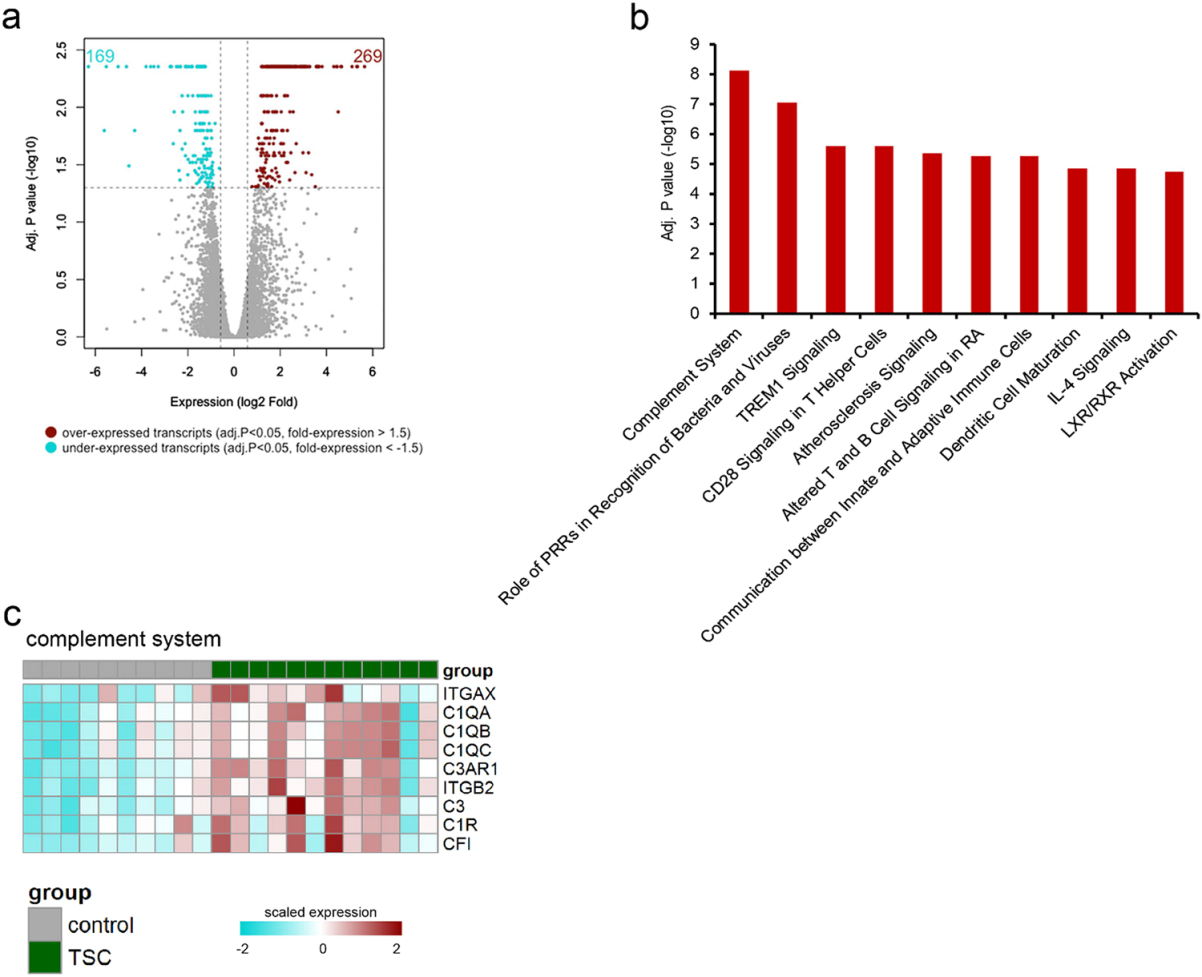
The transcriptome of tuberous sclerosis complex (TSC) cortical tubers determined by using RNA-Seq data (**a**) Volcano plot showing differential expression of genes between TSC tubers (n = 12) and post-mortem control cortex (n = 10). A total of 269 mRNAs were found to be over-expressed and 169 under-expressed in TSC tubers compared to control cortex tissue (**b**) Ingenuity pathway analysis showing major pathways enriched for over-expressed genes in TSC tubers (**c**) Heat map showing genes of the complement system enriched in TSC tubers compared to control cortex. All p-values are BH adjusted.

To characterize the brain transcriptome of TSC subjects, RNA-Seq was performed on mRNA extracted from tubers and normal control brain samples. The analysis included tubers from 12 TSC subjects (10 surgical specimens and 2 autopsy specimens) and 10 age-matched controls without a history of seizures or other neurological disease (See methods and Table 1). On average 23 million paired-end reads were produced per sample. After quality assessment and filtering ~20 million paired-end reads remained per sample, of which ~77% mapped concordantly to the GRCh38 reference genome. Differential gene expression analysis revealed 438 differentially expressed genes (absolute fold-change of >1.5 or <−1.5 and adjusted p-value < 0.05) in the TSC cortical tubers compared to control cortex, 269 of these genes were over-expressed and 169 under-expressed (Fig. 1a). The top 10 over-expressed and under-expressed protein-coding genes (ranked by fold-change) are listed in Table 2. Ingenuity pathway analysis revealed that genes with enhanced expression in tubers were associated with innate and adaptive immune response canonical signaling pathways, including the complement system, triggering receptor expressed on myeloid cells 1 (TREM1) signaling, and CD28 signaling in T helper cells (Fig. 1b). The complement system represented the most significant association, with increased expression of complement C1q A chain (*C1QA*) (3.5-fold), complement C1q B chain (*C1QB*) (3.9-fold), complement C1q C chain (*C1QC*) (3.5-fold), complement component 3 (*C3*) (9.2-fold) and complement C1r subcomponent (*C1R*) (2.2-fold) (Fig. 1c). No canonical signaling pathways were associated with genes that had decreased expression in TSC patients. RT-qPCR targeting a selection of complement system and TREM1 signaling genes validated the RNA-Seq data (Supplementary Fig. 1).

**Table 1:** Summary of clinical characteristics of human specimens.

Patient/Sex/Age (years)	Sample	Brain Region	Mutation	Mutation Location	Epilepsy duration (years)	AED time surgery	ID/ASD
1/F/13	CT	F	*TSC2*	TSC-2 exon 15-26: c.[3232insCACG;1600-75_3232dup13997]	13	LTG, CBZ, CLB	severe/yes
2/M/8	CT	F	*TSC1*	c.2074 A > G	8	LTG, CLB, TPM	mild/yes
3/M/32	CTa	T	*TSC2*	TSC-2 exon 16: c.1839 + 1 G > T	30	PHB, MDZ, LEV	mild/na
4/F/21	CTa	F	*TSC1*	c.2227 C > T	15	VPA	mild/yes
5/M/0.9	CT	T	*TSC1*	TSC-1 exon 21: c.2698 C > T	0.8	CBZ, VPA, VGB	mild/no
6/F/10	CT	F	*TSC2*	c.2721delTinsAG	10	OXC, LTG	mild/no
7/M/47	CT	T	*TSC2*	c.4909_4911delAAG	35	CBZ, CLB	mild/yes
8/M/3	CT	F	*TSC2*	c.3952_3961del10	2	VGB, CBZ, VPA	mild/yes
9/M/10	CT	F	*TSC2*	c.1716 + 1 G > A	8	VPA, CLB	severe/yes
10/F/1	CT	F	*TSC2*	c.4645 C > T	1	VGB	severe/yes
11/F/8	CT	F	*TSC1*	c.1271_1277delGAATGGAinsAT	8	VGB, CBZ, VPA	mild/yes
12/M/3	CT	T	*TSC2*	c.4174 C > T (p.Gln1392X)	3	VGB, LTG, CLB	severe/yes
13/F/0.9	AC	T	—	—	—	—	—
14/F/10	AC	F	—	—	-	—	—
15/F/2.5	AC	F	—	—	—	—	—
16/F/2	AC	F	—	—	—	—	-
17/M/15	AC	F	—	—	—	—	—
18/F/1	AC	T	—	-	—	—	—
19/M/10	AC	F	—	—	—	—	—
20/F/17	AC	T	—	—	—	—	—
21/F/39	AC	F	—	—	—	—	—
22/F/44	AC	F	—	—	—	—	—

Specimens used for: NGS analysis; real-time PCR and *in situ* hybridization. CT, cortical tuber surgery; CTa, cortical tuber autopsy; AC, autopsy control; F: frontal; T: temporal; ID: intellectual disability; ASD: autism spectrum disorder; na: information not available; AED (antiepileptic drugs): CBZ (carbamazepine), CLB (clobazam), LTG (lamotrigine), LEV (levetiracetam), MDZ (midazolam), OXC (oxcarbazepine), PHB (phenobarbital), VGB (vigabatrin)), VPA (valproic acid).

**Table 2:** Top 10 up- and under-expressed protein-coding genes.

Top 10 over-expressed genes						
Gene	Description	Chr. Location	FPKM Control	FPKM TSC	Log2 (Fold-change)	BH adj. p-value
*PLA2G2A*	phospholipase A2 group IIA	chr1:19975430–19980416	0.2128	10.6491	5.6454	0.0044
*PRPH*	peripherin	chr12:49287568–49331731	0.8807	35.6832	5.3404	0.0044
*C21orf62*	chromosome 21 open reading frame 62	chr21:32772099–32893735	0.3509	13.9016	5.3081	0.0044
*CCL4*	C-C motif chemokine ligand 4	chr17:36103589–36105621	1.6944	58.3646	5.1063	0.0044
*CCL4L1*	C-C Motif Chemokine Ligand 4 like 1	chr17:36116176–36439566	2.7548	69.1906	4.6505	0.0044
*LTF*	Lactotransferrin	chr3:46416525–46485234	2.0545	50.5365	4.6204	0.0044
*CCL3*	C-C Motif Chemokine Ligand 3	chr17:36072865–36090169	2.5844	58.6895	4.5052	0.0110
*SLC47A2*	Solute Carrier Family 47 Member 2	chr17:19678276–19718979	0.1928	4.2615	4.4660	0.0044
*CHI3L2*	Chitinase 3 Like 2	chr1:111187058–111243446	7.0674	152.6110	4.4325	0.0044
*CCL3L3*	C-C Motif Chemokine Ligand 3 Like 3	chr17:36116176–36439566	1.9783	39.4745	4.3186	0.0044
**Top 10 under-expressed genes**						
*HEATR6*	Heat Repeat Containing 6	chr17:60040882–60079182	892.769	5.4155	−7.3650	0.0044
*SLC30A2*	Solute Carrier Family 30 Member 2	chr1:26037251–26046160	2.8143	0.0575	−5.6137	0.0159
*SLC22A8*	Solute Carrier Family 22 Member 8	chr11:62934677–63412981	2.8123	0.0609	−5.5293	0.0044
*IL1RL1*	Interleukin 1 Receptor Like 1	chr2:102311501–102398775	19.0343	0.7561	−4.6539	0.0044
*HSPA6*	Heat Shock Protein Family A (Hsp70) Member	chr1:161505429–161678654	68.4820	2.9318	−4.5459	0.0324
*SERPIND1*	Serpin Family D Member	chr22:20707686–20891218	2.6156	0.1329	−4.2992	0.0159
*SLC13A4*	Solute Carrier Family 13 Member 4	chr7:135662487–135748846	9.6643	0.6905	−3.8069	0.0044
*SLC5A5*	Solute Carrier Family 5 Member 5	chr19:17871961–17895174	1.0909	0.0895	−3.6078	0.0044
*DCX*	Doublecortin	chrX:111293779–111412375	10.8126	1.1125	−3.2808	0.0044
*ST8SIA2*	ST8 alpha-N-acetyl-neuraminide alpha-2,8-sialyltransferase 2	chr15:92393827–92468728	3.7169	0.5488	−2.7598	0.0044

To gain a better understanding of potential cell-type specific gene expression related to TSC pathology an independent dataset of single-cell RNA-Seq from neurons, microglia, oligodendrocytes and astrocytes taken from healthy human cortex was analyzed (GSE67835)^23^. Genes from the single-cell RNA-Seq analysis were classified as microglia, oligodendrocyte, astrocyte or neuron specific based on expression values (greater than 10 read-counts in cell type of interest, less than 1 read count in all other cell-types). Of the 269 genes over-expressed in the TSC cortical tubers, 23 were specific to microglia, 3 to oligodendrocytes, 5 to neurons, and 8 to astrocytes (Supplementary Table 1). Amongst the 169 genes under-expressed in the TSC cortical tubers, 6 were specific to neurons, 2 to astrocytes, 1 to microglia and 1 to oligodendrocytes (Supplementary Table 1). A Fisher’s exact test revealed that amongst the significantly over-expressed genes there was significant enrichment for microglia specific (p-value < 2.2e-16) and astrocyte specific (p-value < 0.002) genes, amongst the significantly under-expressed genes there was a suggestive enrichment of neuron specific genes (p-value < 0.05). The 32 genes specific to microglia and astrocytes that were over-expressed in the TSC subjects included the complement system related genes, *C1QA, C1QB, C1QC* and *C4B* (Fig. 1c). We did not observe gene expression differences between important subgroups, that is individuals with *TSC2* versus *TSC1* mutations, or mild versus severe intellectual disability.

### The small non-coding RNA landscape of tuberous sclerosis complex brain tissue

**Figure 2:**
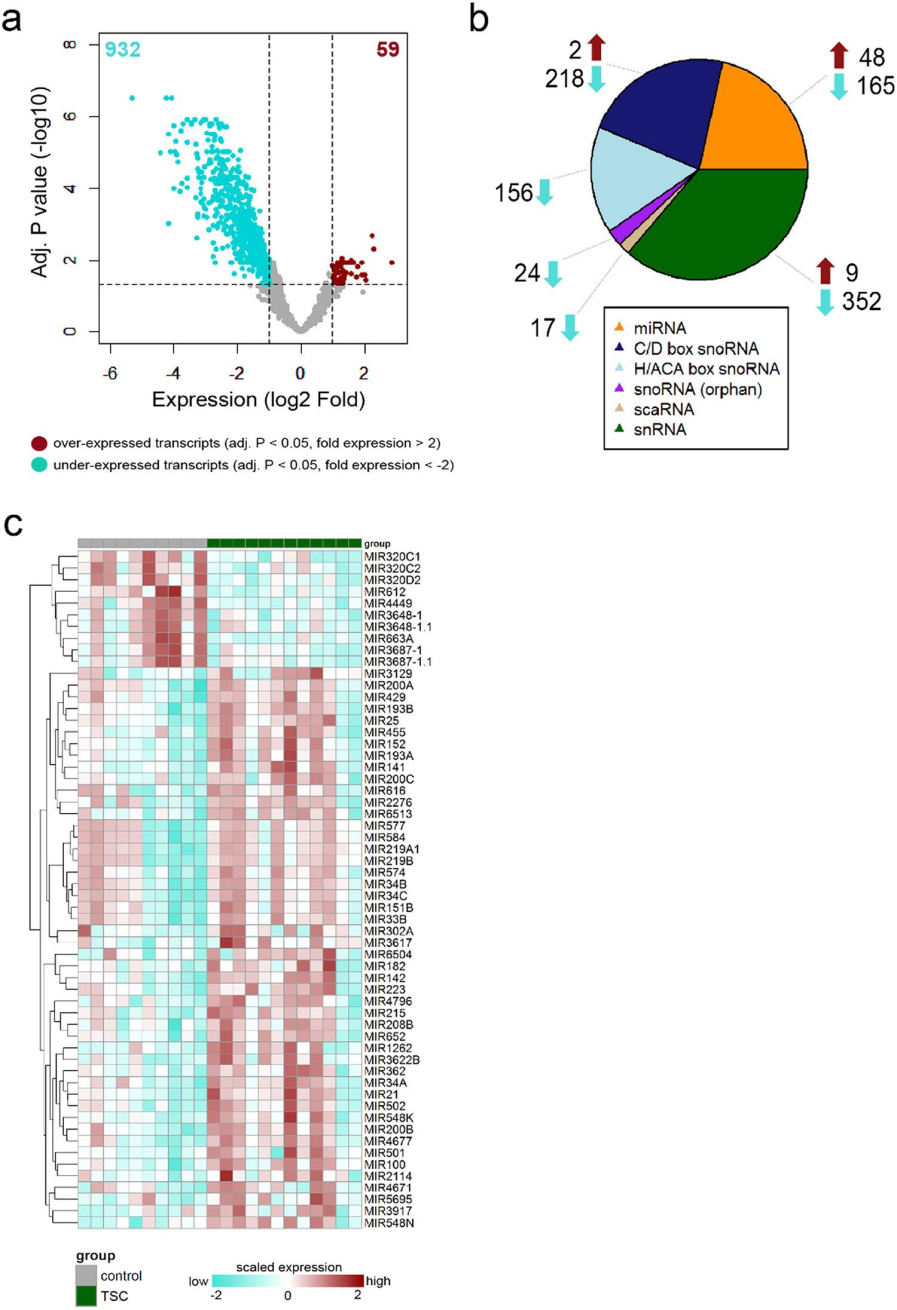
Small RNA landscape of TSC cortical tubers determined by using small RNA-seq data. (**a**) Volcano plot showing differential expression of small RNAs between TSC tubers and post-mortem control cortex. A total of 932 small RNAs were found to be under-expressed and 59 over-expressed in TSC tubers compared to control cortex tissue (**b**) Distribution of various classes of small RNAs among the over- and under-expressed transcripts in TSC cortical tubers (**c**) Heat map showing the expression of the 48 over-expressed and top 10 under-expressed miRNAs in TSC tubers and control cortex.

In order to further explore the brain transcriptome of TSC subjects relative to control subjects we performed small RNA-seq analysis on the same set of RNA samples (Table 1). Each sequencing run produced ~9 million paired-end reads for each sample. After quality assessment and filtering, ~5 million paired-end reads remained for each sample, of which ~82% were mapped to the reference genome (GRCh38). Differential expression analysis of the aligned small RNA transcripts revealed a total of 991 significantly altered transcripts, 59 were elevated and 932 were decreased in TSC cortical tubers compared to controls (Fig. 2a). The differentially expressed small RNAs were not only miRNAs but also other classes (Fig. 2b). The largest class of altered small non-coding RNA was the small nuclear RNA (snRNA). Other classes of altered small RNAs in TSC relative to control patients (in decreasing order) were the C/D box small nucleolar RNAs (snoRNAs), miRNAs, H/ACA box snoRNAs, orphan snoRNA and the small Cajal body RNAs (scaRNAs). Interestingly, the majority of snRNAs, snoRNAs and scaRNAs were under-expressed in TSC cortical tubers compared to control cortex (Fig. 2b; Supplementary Fig. 2). Highly expressed miRNAs in TSC subjects included, miR-34a (3.1-fold), miR-34b (2.6-fold), miR-34c (2.5-fold), miR-302a (2.2-fold), miR-577 (4-fold) and miR-21 (2.9-fold) (Fig. 2c), all members of the miR-34 family were validated using RT-qPCR (Supplementary Fig. 3).

Previously reports of age dependent miRNA expression patterns in the brain and cardiac tissue^24, 25^, notably of miR-34a^26^, coupled with the variability of age in our study cohort motivated us to evaluate the association of age to expression patterns of miR34a and the other members of the miR-34 family members. We found no significant correlation (Pearson’s correlation) between expression patterns of miR-34 family members and age (r < 0.41, p-value > 0.05) (Supplementary Fig. 4).

### Gene co-expression network modules and miRNA targets

**Figure 3:**
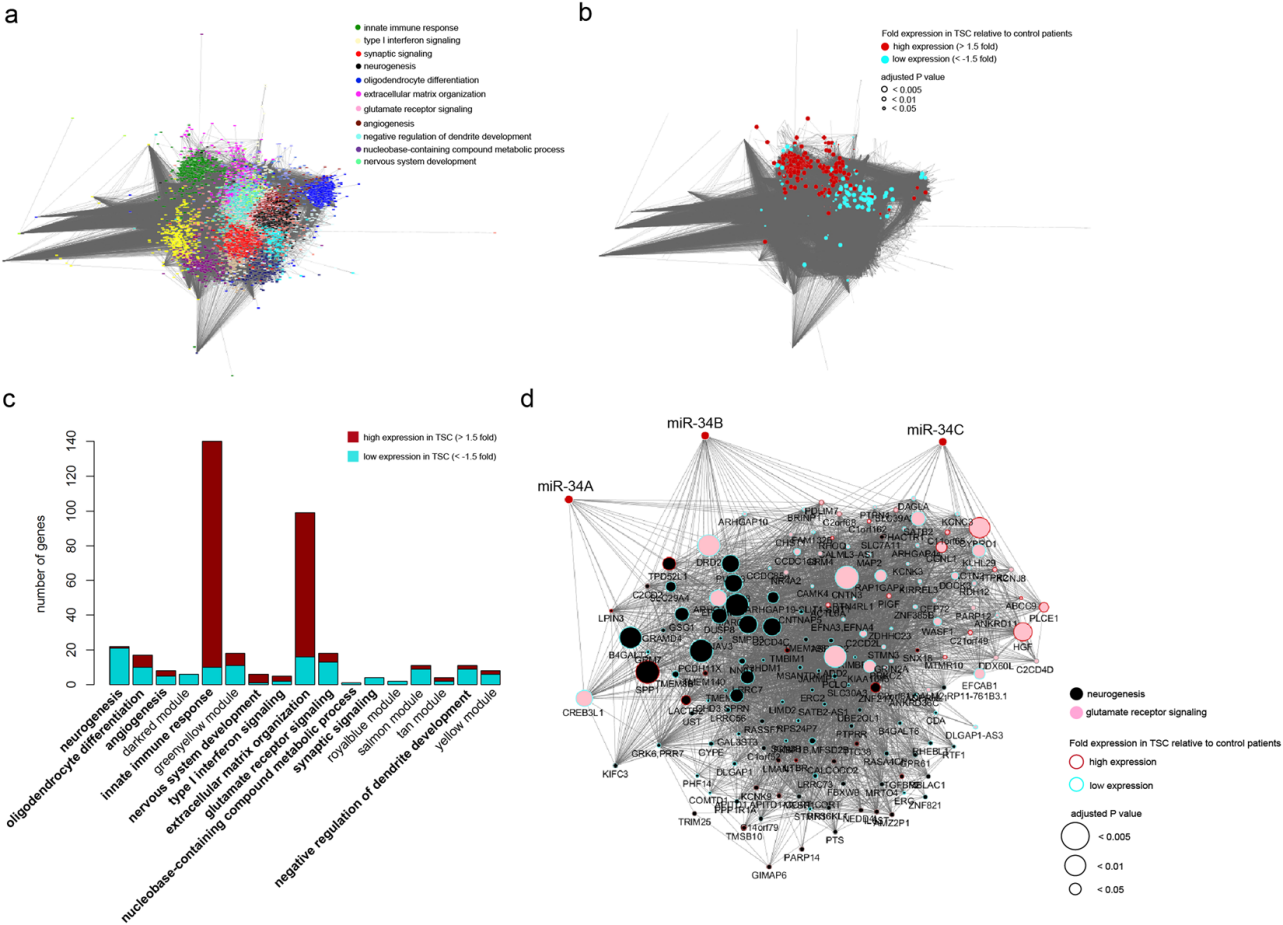
Co-expression network modules and miRNA target predictions. (**a**) Unsupervised weighted gene co-expression network analysis (WGCNA) showing over-represented gene ontology terms in TSC patients. (**b**) Overlay of fold expression (log2 tranformed) of differentially expressed genes between TSC patients and controls on the gene co-expression network. Over-expressed genes predominantly overlap with the innate immune response and extracellular matrix organization modules whereas under-expressed genes overlap with neurogenesis and glutamate receptor signaling module. (**c**) Graphical representation of over- and under-expressed genes enriched within the over-represented co-expression network modules in TSC patients. Over-expressed genes are predominantly associated with the innate immune response and extracellular matrix organization modules whereas under-expressed genes are predominantly associated with neurogenesis and glutamate receptor signaling modules. Only modules that harboured significantly different genes are illustrated. (**d**) The miR-34 family (miR-34A, miR-34B and miR-34C) target multiple targets in the neurogenesis and glutamate receptor signaling modules.

To better understand the organization of the protein coding and small non-coding RNA transcriptome in TSC and control subject brain tissue we applied an unsupervised weighted gene co-expression network approach (WGCNA)^27, 28^. On the basis of a Spearman’s correlation matrix, a weighted network of RNA transcripts was constructed that ensured scale-free topology (see Methods). Unsupervised hierarchical clustering uncovered 21 modules of highly inter-correlating transcripts, each harbouring more than 100 genes (Supplementary Table 2). Modules were analysed for over-representation of gene ontologies and illustrated by an unsupervised Cytoscape yFiles organic layout with each module colour coded (Fig. 3a and Supplementary Table 2). We found 11 modules (out of 21) to be over-represented for various cellular biological pathways, particularly innate immune response (green module), type-I interferon signalling pathway (light yellow module), synaptic signalling (red module), neurogenesis (black module) and extracellular matrix organization (magenta module) modules (Fig. 3a). Overlaying the fold expression (log2 transformed) of significantly differential genes between TSC and control subjects revealed the innate immune response and extracellular matrix organization modules harboured the majority of significantly elevated genes in TSC patients (Fig. 3b and c). Genes with significantly decreased expression in TSC patients were predominantly located in modules attuned to neuronal functions, for example the neurogenesis and glutamate receptor signaling module (Fig. 3b and c). Next, we sought to infer miRNA that potentially target genes within the gene co-expression network. To this aim we firstly leveraged the module eigengene concept (module first principal component) to test for correlations between miRNA expression indices and module expression patterns. Here, we focused our attention on miRNA with elevated patterns of expression in TSC patients relative to controls. In particular, we identified significant indirect negative correlations between the glutamate receptor signalling and neurogenesis modules with expression indices of miR-193, miR-200, and the miR-34 families (Pearson’s correlation) (Supplementary Fig. 5 and Supplementary Table 3). Using the miR-Walk 2.0 database^29^ we identified multiple genes in the neurogenesis and glutamate receptor signalling modules that are predicted targets of miR-34a, miR-34b and/or miR-34c (Fig. 3d, Supplementary Table 4). No overlap was found between the putative miR-34 family targets in the modules neurogenesis and glutamate receptor signalling and any cell-specific genes. Altogether, these results suggest that neurogenesis and glutamate receptor signalling modules may be targeted and, in turn, regulated by specific miRNA families altered in TSC brain tissue.

### Cellular distribution of selected over-expressed miRNAs in TSC cortical tubers and control cortex

**Figure 4:**
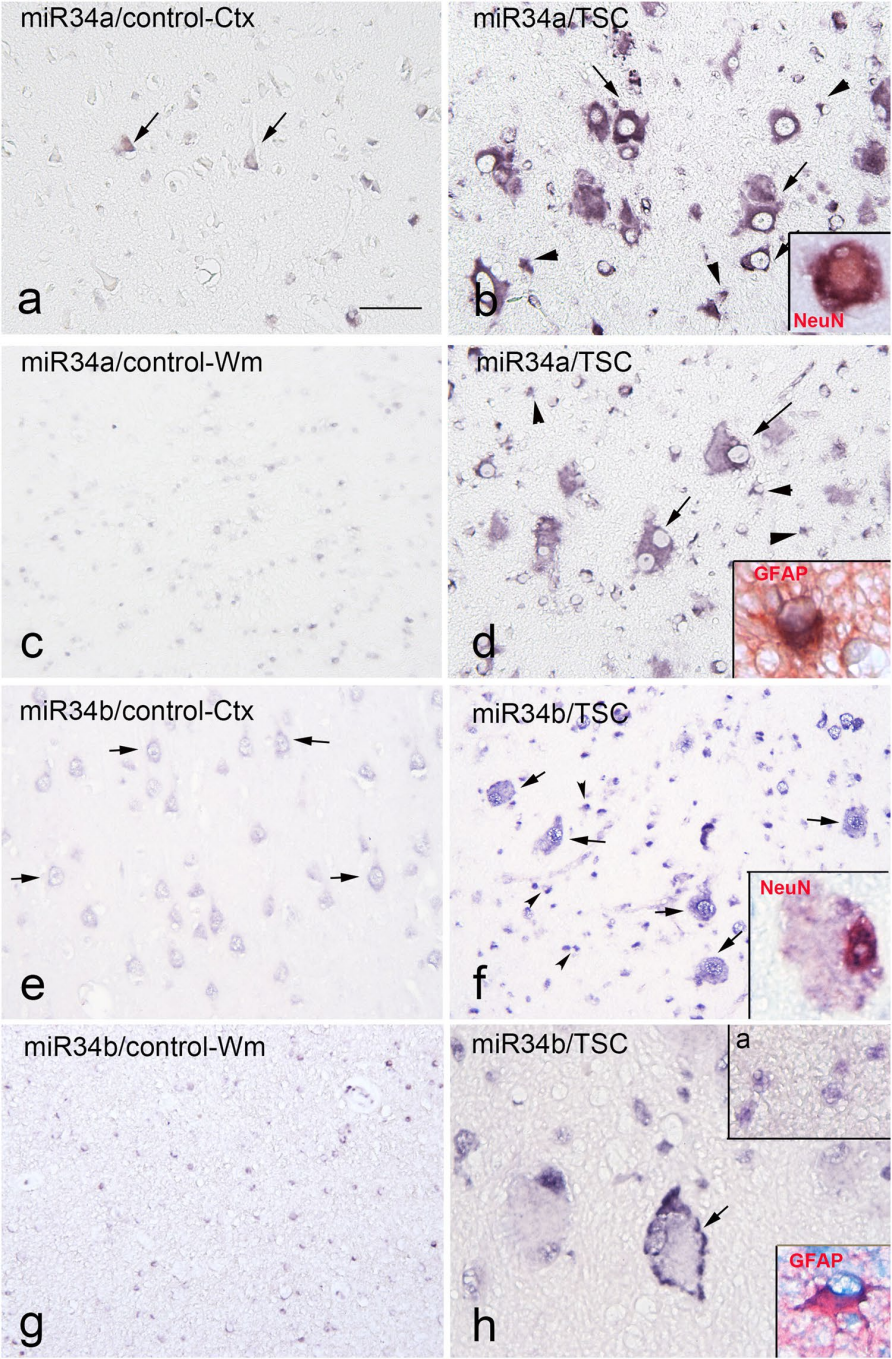
*In situ* hybridization of miR-34a-5p and miR-34b-5p in Tuberous Sclerosis Complex (TSC) cortical tubers. Panels a-d: miR-34a-5p. Control cortex (**a**) shows moderate expression of miR-34a-5p in few neuronal cells (arrows); not detectable expression is observed in control white matter (**c**). Panels b and d (TSC) show strong expression of miR-34a-5p within the dysplastic region with several positive dysmorphic neurons (arrows in **b**) and glial cells (arrowheads in **b**,**d**); insert in b: miR-34b-5p in a NeuN positive cell; insert in d shows colocalization with GFAP. Expression of miR-34a-5p is also detected in giant cells within the tuber white matter (arrows in **d**). Panels e-h: miR-34b–5p. Control cortex (**e**) shows moderate expression of miR-34b-5p in neuronal cells (arrows); very low expression is observed in control white matter (**g**). Panels f and h (TSC) show expression of miR-34b-5p within the dysplastic region with several positive dysmorphic neurons [arrows; insert in **f**: miR-34b-5p in a NeuN positive cell] and glial cells [arrowheads in **f** and insert (**a**) in **h**; insert (**b**) in **h** shows colocalization with GFAP]; arrow in **h** shows a positive giant cell within the tuber white matter. Scale bar in **a**: (**a**–**g**), 80 µm; (**h**), 40 µm. [arrows in **h** and insert (**a**) in **h**, white matter]. Scale bar in **a**: (**a**,**e**,**f**): 160 µm; (**b**–**d**,**g**,**h**) 80 µm.

Thus far our findings suggest that typical neuronal cellular pathways were predicted targets of specific miRNA family members. Considering our analysis was centered upon complex brain tissue, which encompasses multiple cell-types, we here sought to gain insight into the cell-specific patterns of selected miRNA expression in TSC and control brain tissue, that is, the miR-34 family members, miR-34a and miR-34b. In line with our RNA-seq data, *in situ* hybridization targeting miR-34a-5p and miR-34b-5p showed low expression in control cortex for both miRNAs (Fig. 4a,e) and high expression in TSC tubers, specifically dysmorphic neurons, giant cells and in cells with astroglial morphology (Fig. 4b,d,f,h). Double labeling confirmed the expression of these miRNAs in NeuN (neurons) – and GFAP (astrocytes)- positive cells in TSC cortical tuber specimens (Fig. 4).

### Overexpression of miR-34b increases expression of *IL1B* in human foetal astrocytes

To study the impact of miR-34a-5p and miR-34b-5p overexpression in foetal astrocytes two different assays were performed. First, in an attempt to induce miR-34a-5p and miR-34b-5p expression foetal astrocytes were stimulated with IL1β. No increase in the expression levels of miR-34a-5p and miR-34b-5p due to IL1 β were observed. Subsequently, the foetal astrocytes were transfected with a miR-34a-5p and a miR-34b-5p mimic. Foetal astrocytes transfected with the miR-34b-5p mimic had an associated up-regulation of *IL1β* (~4-Fold, p-value < 0.03) and the inflammatory marker *IL6* (~2.3-fold, p-value < 0.03), (Supplementary Figure 6). Conversely, no increase in *IL1β* and *IL6* levels was seen after transfection with the miR-34a-5p mimic, and a down regulation of *COX2* (~1.5-fold, p-value < 0.03) was observed. This suggests that an overexpression of miR-34b-5p in astrocytes could activate an inflammatory response in astrocytes.

### miR-34b modulates neurite outgrowth in mouse hippocampal neuronal cultures

**Figure 5:**
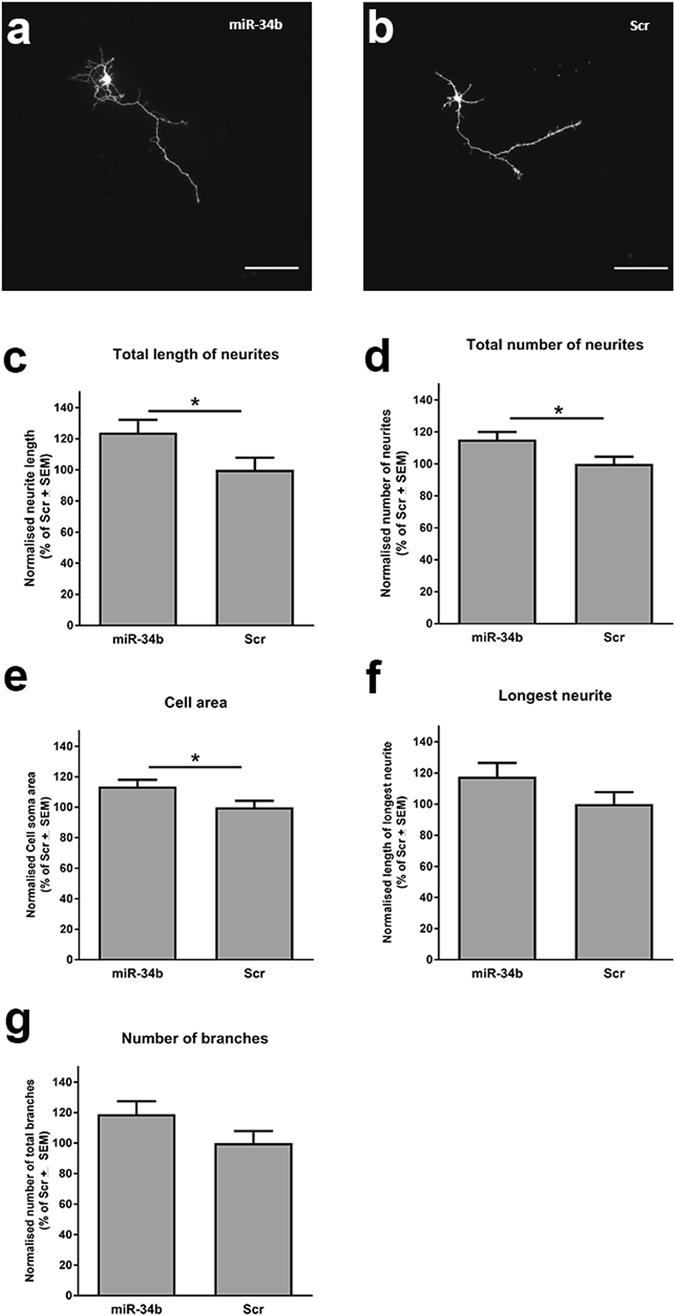
Neurite outgrowth modulated by transfection with miR-34b-5p in mouse hippocampal neurons (**a**,**b**) Representative images of mouse hippocampal dissociated neurons cotransfected with GFP vector and mimics for miR-34b-5p (miR-34b) or NC-1 (negative control; Scr); Scale bar, 100 µm. (**c**–**f**) Graphical representation of neurite outgrowth analysis using the NeuroMath software showing (**c**) total length of neurites (**d**) total number of neurites (**e**) cell area (**f**) longest neurite and (**g**) total number of branches. Student’s t-test: *p < 0.05; n = 3 experiments, 3 independent transfections each experiment.

To further explore the capacity of miR-34 family members to modulate neuronal characteristics, as predicted by our miRNA-to-module interaction framework, we selected miR-34b for further functional studies *in vitro*. In particular we investigated the impact of miR-34b overexpression on neurite outgrowth in a mouse hippocampal neuron model. Primary mouse neuronal cultures prepared from the hippocampi of postnatal day 0 (P0) C57Bl/6 mice were transfected at 1 day *in vitro* with either the miR-34b-5p mimic or the miRNA mimic negative control (Scr) and analyzed for neurite outgrowth at 4 days *in vitro*. Neurons transfected with miR-34b-5p mimic showed an increased number of longer neurites as compared to Scr transfected cultures (Fig. 5a,b). Quantification using the NeuroMath software showed a significant increase in the total length (Fig. 5c), number of neuritis (Fig. 5d) and an increase in soma size (Fig. 5e) of the miR-34b-5p mimic transfected neurons compared to Scr transfected cultures.

## Discussion

In this study we report a comprehensive analysis of the brain transcriptome in TSC patients relative to control subjects. Evaluation of both the protein coding and small non-coding RNA by RNA-seq revealed substantial alterations in TSC brain tissue. In particular, TSC cortical tubers had elevated expression of genes involved in innate immune pathways, such as complement system, concomitant with decreased expression of genes predominantly associated with neuronal cellular pathways, including neurogenesis and glutamate receptor signaling. A variety of small non-coding RNA transcripts, including miRNAs, were also significantly altered in TSC cortical tubers relative to controls. Application of a gene co-expression network approach and miRNA-to-target predictions allowed for the identification of functional modules of the brain transcriptome potentially modulated by miRNAs. Based on these predictions miR-34 family members, notably miR-34b, were assessed by functional *in vitro* studies and shown to possess a capacity to modulate neurite outgrowth and to activate an inflammatory response in astrocytes.

Heightened expression of genes involved in innate and adaptive immune pathways, such as complement system, TREM1 signaling and CD28 signaling was an overarching feature of the TSC cortical tuber protein coding transcriptome. These data extend on our previous study, a genome wide microarray analysis of a small cohort of TSC cortical tubers and four controls demonstrating up-regulation of genes related to the inflammatory response, including complement factors, chemokines and several cytokines^14^. Others have also shown differential inflammatory gene expression in TSC cortical tubers; albeit by targeted RT-qPCR and immunocytochemistry^14, 30, 31^, indicating that our data on mRNA expression changes in TSC cortical tubers were consistent across different sample cohorts and studies. The presence of biologically active fragments of C3 (C3c and C3d); and C4 (C4b) in TSC cortical tubers indicates that the activation of the complement cascade may be a key driver of the inflammatory response consistently observed in the TSC brain^14, 30, 31^. The complement cascade has been implicated in seizure generation and progression in numerous experimental epilepsy models^32–36^, human temporal lobe epilepsy^37^, and in epileptogenic glioneuronal tumors^38^. Also, our data suggest a role for TREM-1 signaling in TSC. TREM-1 can amplify Toll like receptor signaling, thereby increasing the production of a wide range of cytokines and chemokines^39, 40^. Interestingly, experimental studies support the role of these pathways in seizures^35, 41, 42^, suggesting that drugs targeting the TREM-1 signaling may have potential therapeutic benefit for seizures in TSC. Evaluation of fetal TSC brain revealed an activation of the innate and adaptive immune response early during brain development^43^ suggesting that the induction of this pathway could be intrinsic to the TSC developmental pathology and linked to the deregulation of the mTOR pathway^44–46^. Accordingly, a recent study shows over-activation of pro-inflammatory signaling pathways in astrocytes before epilepsy onset in a mouse model of TSC, pointing to the role of mTOR-mediated inflammatory mechanisms in TSC^47^. Amongst the significantly over-expressed genes in TSC subjects there was enrichment for microglial and astrocyte specific genes. Both microglia and astrocytes are involved in inflammation driven processes throughout the brain^48, 49^, suggesting that the TSC inflammatory processes may be driven by activation of microglia and astrocytes. Understanding whether changes in innate and/or adaptive immune gene expression represent the cause or the consequence of chronic pharmacoresistant seizure activity is extremely difficult since epilepsy surgery targeting cortical tubers or autopsy is performed several months or years after the start of seizures. Moreover, a comparison with non-epileptogenic tubers could not be performed, since this tissue is not resected during epilepsy surgery and infrequently available at autopsy. Nevertheless these findings provide further evidence for the activation of both the innate and adaptive immune response cascades in the pathophysiology of TSC.

Besides the protein coding transcriptome we also mapped the small non-coding RNA in TSC brain tissue relative to controls. We found that not only miRNA, extensively investigated as epigenetic effector molecules, were significantly altered in the TSC brain transcriptome but also other species of small RNA, notably snoRNAs, snRNAs and scaRNAs. These small RNA species represent an ancient group of non-coding RNA molecules, which in particular to snoRNA (including the H/ACA box and C/D box categories) may spread throughout the genome by retrotransposition, representing a new family of mobile genetic elements^50, 51^. These recently discovered small RNA have been shown to modify the chemical properties of ribosomal RNAs and transfer RNAs, impacting on mRNA translation, RNA silencing, alternative splicing^52–54^, as well as methylation and pseudouridylation of spliceosomal RNAs in the Cajal bodies of the nucleus^55, 56^. Notably, the disruption of snoRNA expression and/or function has been implicated in neurodevelopmental disorders such as Prader-Willi syndrome and autism spectrum disorders^57, 58^. These as yet under-explored classes of small non-coding RNA certainly warrant further investigation in TSC pathogenesis.

Through the use of an unsupervised systems-based computational technique, that is gene co-expression network construction, we identified multiple modules of tightly inter-correlating protein coding genes. This approach allowed for the construction of a holistic transcriptome model, thus not only those functions biased towards the response in TSC relative to control subjects (supervised analysis). This aspect is supported by the finding of modules enriched for genes involved in neuronal attuned biological functions, such as neurogenesis and glutamate receptor signalling, which would otherwise have been missed. Moreover, WGCNA has emerged as an important tool in integrative bioinformatics/genomics^59^. We reasoned that since miRNA are known to target and influence the expression of multiple genes involved in shared and distinct cellular processes, a modular approach may uncover functional units of distinct biological functions in the brain transcriptome potentially modulated by miRNA, rather than considering single transcripts. In so doing, we uncovered the miR-34 family (miR-34a, miR-34b and miR-34c) as predicted modifiers of neurogenesis and glutamate receptor signalling transcriptional output. These predictions were further tested experimentally using a miR-34b-5p overexpression system in mouse hippocampal neurons, demonstrating that miR-34b-5p can modulate neurite outgrowth. Reduced neurogenesis during chronic epilepsy could result in loss-of-function deficits and contribute to common TSC comorbidities such cognitive impairment or depression^60, 61^. Also, previous findings have demonstrated abnormal accumulation of extracellular glutamate occurring in human epileptogenic tissue, which is hypothesized as a key factor in recurrent seizures and neuronal death^62^. Mouse models have also shown that controlling seizures by antiepileptic drugs (AEDs) may act via alterations in brain glutamate dehydrogenase activity as well as the brain transcriptome including miRNAs^63, 64^. Both miR-34a and miR-34b have been previously identified as overexpressed miRNAs in tubers^15^, as well as key tumour suppressors downstream of the p53 pathway and have been suggested as potential targets of therapy in several cancers^65, 66^. Members of the miR-34 family have also been shown to regulate key pathways in neurodevelopment and cortical neurogenesis, such as the Notch^67–69^ and the Wnt signaling pathway^70, 71^. More specifically, it appears that miR-34a plays a role in neuronal differentiation^72–77^, where overexpression of miR-34a in mouse neural stem cells impairs both neuronal differentiation and synapse function^74–78^. Moreover, a recent study has demonstrated targeting of the *TSC1* 3′ UTR by miR-34a, supporting the role of this miRNA in tuber pathology^15^. In this particular study epileptogenic tubers were compared to adjacent non-tuber tissue indicating that elevated expression of miR-34 members may indeed represent an important feature of tuber physiology rather than an effect of AED treatment.

Our study has limitations. TSC patients received individually tailored AED regimens contrary to autopsy control subjects. Moreover, AEDs were given in multiple combinations, which precludes proper evaluation of the effects of AEDs on gene expression alterations in TSC tubers.

In conclusion, our study provides a comprehensive analysis of the coding and small non-coding transcriptional landscape of TSC cortical tubers. The TSC transcriptomic network reflects the prominent activation of both innate and adaptive immune response characteristic of cortical tubers, with identification of key pathways such as complement system, TREM1 and CD28 signaling. Notably, the under-expressed genes were linked to neurogenesis and glutamate receptor signaling. We identified a variety of small RNA molecules, including miRNA, snoRNA, snRNA and scaRNA, differentially expressed in TSC. Moreover, our study predicts an important role for the miR-34 family (miR-34a, miR-34b and miR-34c) as modifiers of neurogenesis and glutamate receptor signaling in TSC, which may potentially provide an epigenetic-driven therapeutic tool for epilepsy and cognitive disabilities in TSC.

## Methods

### Human specimens

The cortical specimens from TSC and control patients included in this study were selected from the archives of the departments of neuropathology of the Academic Medical Center (AMC, University of Amsterdam, The Netherlands), the University Medical Center Utrecht (UMCU, The Netherlands), Motol University Hospital (Prague, Czech Republic) and Medical University Vienna (MUV, Austria). Informed consent was obtained for the use of brain tissue and for access to medical records for research purposes. We evaluated 12 TSC specimens (10 surgical specimens and 2 autopsy specimens) from whom we obtained anatomically well-preserved neocortical tubers tissue and sufficient clinical data. All patients fulfilled the diagnostic criteria for definite TSC^79, 80^. The surgical cases had pharmacologically intractable epilepsy (daily seizures) and underwent extensive pre-surgical evaluation^6^. Patient characteristics are summarized in Table 1. The following clinical data were extracted from medical records: TSC1/TSC2 mutation status, gender, localization of the resected area, age at seizure onset, duration of active epilepsy, antiepileptic drug (AED) management at time of surgery and presence/absence of intellectual disability (ID; Mild = IQ 55–70; Moderate = IQ 40–55; Severe IQ 25–40) and autism. The majority of TSC patients harbored *TSC2* gene mutations (66.6%; Table 1). The tissue specimens were age-matched with the controls (p-value > 0.86, two-sided t-test). Using a principal variance component analysis (PVCA) brain region and gender were shown to contribute minimally to the overall variation of the RNA-Seq results.

Perituberal frozen material was available for only 3 out of 10 TSC cases examined and two samples did not pass the minimal quality requirements; thus analysis of perituberal tissue was not performed. Histologically normal cortex was obtained at autopsy from 10 controls without a history of seizures or other neurological diseases (cause of death was acute cardiorespiratory failure; Table 1). We acknowledge that the choice of the control material is extremely difficult in human studies, particularly in case of pathologies affecting young patients, which limits the number of cases suitable for gene expression studies. We were fortunate to obtain the human postmortem from young controls, as well as autopsy TSC samples.

Tissue was obtained and used in accordance with the Declaration of Helsinki and the AMC Research Code provided by the Medical Ethics Committee and approved by the science committee of the UMC Utrecht Biobank. The local ethical committees of all participating centers gave permission to undertake the study.

### Tissue preparation

Cortical specimens from control and TSC patients were snap frozen in liquid nitrogen and stored at −80 °C until use for RNA isolation. Additional tissue was fixed in 10% neutral buffered formalin and embedded in paraffin. Paraffin-embedded tissue was sectioned at 5 μm, mounted on pre-coated glass slides (Star Frost, Waldemar Knittel GmbH, Brunschweig, Germany) and used for *in situ* hybridizations and immunocytochemistry. One representative paraffin block per case was sectioned and processed for hematoxylin and eosin (HE) as well as for immunocytochemical staining for a number of neuronal and glial markers to confirm the diagnosis.

### RNA isolation

Frozen tissue material was homogenized in Qiazol Lysis Reagent (Qiagen Benelux, Venlo, The Netherlands). The total RNA including the miRNA fraction was isolated using the miR-Neasy Mini kit (Qiagen Benelux, Venlo, the Netherlands) according to manufacturer’s instructions. The concentration and purity of RNA were determined at 260/280 nm using a Nanodrop 2000 spectrophotometer (Thermo Scientific, Wilmington, DE, USA) and RNA integrity was assessed using a Bioanalyser 2100 (Agilent). Samples required an RNA integrity number (RIN) greater than 6.0 for use in down-stream sequencing.

### RNA-seq library preparation and sequencing

Library preparation and sequencing were completed at ServiceXS, Plesmanlaan 1D, 2333 BZ, Leiden, Netherlands. The Illumina (San Diego, California, USA) mRNA-Seq and TruSeq Small RNA-Seq sample preparation kits were used to prepare sequencing libraries of mRNA and small RNA, respectively. Briefly, mRNA was selected by oligo-dT magnetic beads, fragmented and subjected to cDNA synthesis. Sequencing adapters were ligated to the cDNA fragments, followed by PCR amplification. Small RNA samples were processed by size exclusion gel electrophoresis subsequent to sequencing adaptor ligation. After gel excision and digestion, sequences were amplified by PCR. Clustering and DNA sequencing was performed using the Illumina cBot and HiSeq 2500. Each library was subjected to paired-end sequencing, producing reads 125 nucleotides in length.

### Bioinformatics analysis of RNA-seq data

Read quality was assessed using FastQC v0.11.2 (Babraham Institute, Babraham, Cambridgeshire, UK), and Trimmomatic v0.33 was used to trim and filter reads of low quality^81^. Low quality leading and trailing bases were removed from each read, a sliding window trimming using a window of 4 and a phred33 score threshold of 20 was used to assess the quality of the body of the read. Reads that dropped below 36 and 18 nucleotides, respectively in our mRNA and small RNA datasets, as well as sequence reads lacking both forward and reverse orientations were excluded from further analysis.

Next, paired-end reads were aligned to the human reference genome (GRCh38) with TopHat2 v2.0.13 using the default settings^82^. No mismatches between the small RNA trimmed reads and reference genome were allowed. The aligned mRNA reads were assembled into individual transcripts and the abundance of each transcript was estimated using Cufflinks v2.2.1^83^. For mRNA sequence data, expression level was calculated as fragments per kilobase of exon per million fragments mapped (FPKM). The Cufflinks transcript assembly was guided using the reference annotation file Gencode v21^84^. The transcript assemblies from each sample were then assembled into a single unified transcript catalog using Cuffmerge^83^. Finally, the merged transcript file along with the original alignment files produced from Tophat were analyzed using Cuffdiff. Libraries were quantile-normalized and differential expression analysis was performed considering genes with FPKM >1 in at least one of the sample groups. Small RNA count data were normalized (per 1 × 10^6^ reads) and differential expression analysis between TSC and control patients was done by means of the limma method (version 3.14.4)^85^. Throughout Benjamini-Hochberg (BH)^86^ multiple comparison adjusted probabilities (P < 0.05) defined significance. Ingenuity Pathway Analysis (Ingenuity Systems IPA, www.ingenuity.com) was used to identify the associating canonical signaling pathways stratifying genes by over- and under-expressed patterns. The Ingenuity gene knowledgebase was selected as reference and human species specified. All other parameters were default. Fisher’s exact test BH-adjusted probabilities (P < 0.05) defined significance.

### Integrative bioinformatics

The mRNA sequence transcriptome was analyzed by means of a weighted gene coexpression network approach^87, 88^. A pair-wise Spearman’s correlation matrix of the top 10000 most variable unique genes (ranked by median absolute deviation) was transformed into an adjacency matrix by using a soft power function to ensure scale-free topology^89^. The adjacency matrix was further transformed into a topological overlap matrix to enable the identification of modules (clusters) of highly correlating genes by implementing a dynamic tree cut algorithm^90, 91^. Thus, each module representing a cluster of co-regulated genes with a distinct expression pattern from other identified modules. In order to define module “driver” genes we made use of the module eigengene concept, defined as the first principal component of the module expression matrix, and, the module membership measure, k^90, 91^. Significantly different miRNA expression indices between TSC and control patients were interrogated for their predicted interations with module-specific genes by means of the miR-Walk atlas, specifying the miR-Walk algorithm, of gene-miRNA-target interactions^92^. We selected for human species annotations and considered 3’UTR interactions as well as a minimum seed length equating to 7. All other parameters were default. Correlation analysis between miRNA profiles and module eigengenes was performed by Pearson’s method. Significant correlations were demarcated by p < 0.05.

### Single cell RNA-seq analysis

Single cell RNA-Seq data sets produced by the Quake laboratory (Stanford University, CA), were retrieved from NCBI’s Gene Expression Omnibus (GEO) (accession no. GSE67835)^93^. Data was retrieved for four different cell types; neurons, oligodendrocytes, microglia and astrocytes, all from healthy human cortex. Three biological replicates for each cell type were used, giving 12 samples in total. Sequence reads were trimmed and filtered using FastQC v0.11.2 and Trimmomatic v0.33 as aforementioned. Paired-end reads were aligned to the human reference genome (GRCh38) with TopHat2 v2.0.13 using the default settings^82^. Next, the number of reads that mapped to each gene in the genome was calculated using featureCounts from the SubRead package^94^. The GRCh38 reference annotation file Gencode v21 was used as an input for featureCounts^84^. Data analysis and manipulation was performed in R (version 3.2.4). The count matrix was normalized using the R package DESeq2^95^.

### DNA analysis

TSC1 and TSC2 mutation analysis was performed by sequence analysis of all coding exons and exon/intron boundaries. Mutations are described according to HGVS nomenclature (Accession number NM_000548.3)^96^.

### *In situ* hybridization

*In situ* hybridization (ISH) for miR-34a-5p and miR-34b-5p were performed on 5 μm thick FFPE tissue using 5′ - 3′ double digoxygenin (DIG)-labeled probes as described previously^97, 98^. The probe sequences used were: miR-34b-5p: 5′ DIG-AugGcaGugGagTuaGugAuuG-DIG;from Ribotask ApS (Odense, Denmark) and miR-34a-5p: 5′ DIG- AcaAccAgcTaaGacAcuGccA-DIG (Exiqon A/s, Vedbaek, Denmark) (capital letter = LNA modification, small letter = 2-o-methyl modification). Briefly, after the sections were deparaffinized and heat-treated to undo protein crosslinks (10 min at 120 °C in a pressure cooker), the probes were hybridized at 56 °C for 1 h. The hybridization was detected with an alkaline phosphatase (AP)-labeled anti-DIG antibody (Roche Applied Science, Basel, Switzerland). NBT (nitro-blue tetrazolium chloride)/BCIP (5-bromo-4-chloro-3′-indolyphosphate p-toluidine salt) was used as chromogenic substrate for AP. Negative controls sections were without probes and without primary antibody. For the double-staining, combining immunohistochemistry with ISH, the sections were first processed for ISH and then processed for immunohistochemistry with glial fibrillary acidic protein (GFAP, astrocyte marker; monoclonal mouse, Sigma, St. Louis, Mo, USA; 1:4000), NeuN (neuronal nuclear protein; mouse clone MAB377; Chemicon, Temecula, CA, USA; 1:2000), and HLA-DP/DQ/DR (microglial marker, mouse clone CR3/43; Dako; 1:100). Signal was detected using the chromogen 3-amino-9-ethylcarbazole (Sigma-Aldrich, St. Louis, MO, USA).

### Quantitative reverse-transcription PCR analysis (RT-qPCR)

miRNAs (miR-34a-5p, miR-34b-5p, miR-34c-5p, miR-302a-3p, miR-21-5p and the reference small nuclear RNAs, Rnu6B and Rnu44) expression was analyzed using Taqman micro RNA assays (Applied Biosystems, Foster City, CA). cDNA was generated using Taqman MicroRNA reverse transcription kit (Applied Biosystems, Foster City, CA) according to manufacturer’s instructions and the PCRs were run on a Roche Lightcycler 480 thermocycler (Roche Applied Science, Basel, Switzerland). Quantification of data was performed using the computer program LinRegPCR in which linear regression on the Log (fluorescence) per cycle number data is applied to determine the amplification efficiency per sample^99, 100^. The starting concentration of each specific product was divided by the starting concentration of reference genes (geometric mean of Rnu6B and Rnu44 values) and this ratio was compared between groups.

To evaluate expression of miRNA targets and inflammation-related genes, 2.5 µg of total RNA was reverse-transcribed into cDNA using oligodT primers. PCR primers (Eurogentec, Belgium) were designed using the Universal ProbeLibrary of Roche (https://www.roche-applied-science.com) on the basis of the reported cDNA sequences (Supplementary Table 5). For each PCR, a mastermix was prepared on ice, containing per sample: 1 µl cDNA, 2.5 µl of 2x SensiFAST^TM^ SYBR Green Reaction Mix (Bioline Inc, Taunton, MA, USA), 0.4 µM of both reverse and forward primers and the PCRs were run on a Roche Lightcycler 480 thermocycler (Roche Applied Science, Basel, Switzerland). Quantification of data was performed as described for the Taqman PCR and the starting concentration of each specific product was divided by the geometric mean of the starting concentration of reference genes (*EF1A*, *C1orf43* and *SNRPD3*) and this ratio was compared between patient/control groups.

### Human astrocyte-enriched cell cultures

Primary foetal astrocyte-enriched cell cultures were prepared from tissue collected from donors from whom a written informed consent for the use of the material for research purposes has been obtained by the Bloemenhove Clinic (Heemstede, The Netherlands). Cell isolation was performed as described elsewhere^98^. Cell cultures were stimulated with human recombinant (r) IL1β (Peprotech, NJ, USA; 10 ng/ml) for 24 hours. For transfection Foetal astrocyte cultures were transfected with either mirVana^TM^ miR-34b-5p miRNA mimic or mirVana™ miRNA Mimic, Negative Control #1 (both from ThermoFisher Scientific, Landsmeer, Netherlands) at a final concentration of 50 nM using Lipofectamine® 2000 transfection reagent (ThermoFisher Scientific, Landsmeer, Netherlands). Cells were harvested after 24 hours of stimulation/transfection for RNA isolation and RT-qPCR (miR-34a-5p, miR-34b-5p, *IL1β*, *IL6*, *COX2*).

### Primary mouse hippocampal neuron cultures, immunofluorescent staining and neurite growth analysis

Primary mouse hippocampal neuron cultures were prepared from postnatal day 0 (P0) C57BL/6 mouse brains. Cells were plated on 15 mm coverslips coated with poly-D-lysine (20 µg/ml) and laminin (40 µg/ml) in 24-well plates at a density of 100,000 cells/well. The cultures were grown in Neurobasal medium (NB) supplemented with B27, 0.35 mM HEPES, 200 mM L-glutamine, 14.3 mM β-mercaptoethanol and penicillin/streptomycin. Cultures were co-transfected at 1 day *in vitro* with miR-34b-5p miR-Vana mimic (Applied Biosystems, Life Technologies Europe BV, Bleiswijk, Netherlands) or the miR-IDIAN miRNA mimic negative control #1 (Dharmacon, GE Healthcare Europe, Eindhoven, the Netherlands) and a green fluorescent protein (GFP) vector at 50 pmol/well using Lipofectamine®-2000 (Thermo Fisher Scientific) as transfection reagent for 1 hour at 37 °C and 5% CO2. Cultures were fixed with 4% paraformaldehyde/4% sucrose in phosphate-buffered saline (PBS) at 4 days *in vitro* for 20 minutes and washed three times with PBS for 30 min at room temperature. The cultures were blocked with 0.1% PBS-Triton X-100 buffer with 3% Normal Goat serum and then incubated with primary antibodies in the blocking solution overnight at 4 °C. Subsequently, neurons were washed three times in PBS and incubated with AlexaFluor-conjugated secondary antibodies in the blocking buffer for 2 hr at RT. They were then washed 2 times with PBS and incubated with DAPI (nuclear) staining. After final washes with PBS for 20 min, the neurons were mounted on slides with Fluorsave (Merck Millipore) mounting medium. Images were obtained at 20x magnification with epifluoroscent Axioscope A1 (Zeiss) microscope. Neurite outgrowth was analyzed using NeuroMath developed at Weizmann Institute, Israel.

### Data availability

The datasets generated during and/or analyzed during the current study are available at the following repositories:

#### Primary

RNA-seq and small RNA-Seq data from TSC and control patients: Sequence data has been deposited at the European Genome-phenome Archive (EGA), which is hosted by the EBI and the CRG, under the accession number: EGAS00001002485.

Further information about EGA can be found on https://ega- archive.org “The European Genome-phenome Archive of human data consented for biomedical research”(http://www.nature.com/ng/journal/v47/n7/full/ng.3312.html).

#### Secondary

Single cell RNA-seq data: NCBI’s Gene Expression Omnibus (GEO) under the accession number: GSE67835 Web link: www.ncbi.nlm.nih.gov/geo/query/acc.cgi?acc=GSE67835.

## Electronic supplementary material

Supplementary PDF File
