# Author Correction: Coding and small non-coding transcriptional landscape of tuberous sclerosis complex cortical tubers: implications for pathophysiology and treatment

**DOI:** 10.1038/s41598-022-20109-7

**Published:** 2022-09-14

**Authors:** James D. Mills, Anand M. Iyer, Jackelien van Scheppingen, Anika Bongaarts, Jasper J. Anink, Bart Janssen, Till S. Zimmer, Wim G. Spliet, Peter C. van Rijen, Floor E. Jansen, Martha Feucht, Johannes A. Hainfellner, Pavel Krsek, Josef Zamecnik, Katarzyna Kotulska, Sergiusz Jozwiak, Anna Jansen, Lieven Lagae, Paolo Curatolo, David J. Kwiatkowski, R. Jeroen Pasterkamp, Ketharini Senthilkumar, Lars von Oerthel, Marco F. Hoekman, Jan A. Gorter, Peter B. Crino, Angelika Mühlebner, Brendon P. Scicluna, Eleonora Aronica

**Affiliations:** 1 Department of (Neuro) Pathology, Academic Medical Center, University of Amsterdam, Amsterdam, The Netherlands; 2 GenomeScan BV, Leiden, The Netherlands; 3 Department of Pathology, University Medical Center Utrecht, Utrecht, The Netherlands; 4 Department of Neurosurgery, Rudolf Magnus Institute for Neuroscience, University Medical Center, Utrecht, The Netherlands; 5 Department of Pediatric Neurology, Rudolf Magnus Institute for Neuroscience, University Medical Center, Utrecht, The Netherlands; 6 Department of Pediatrics, Medical University Vienna, Vienna, Austria; 7 Institute of Neurology, Medical University Vienna, Vienna, Austria; 8 Department of Pediatric Neurology, 2nd Faculty of Medicine and Motol University Hospital, Prague, Czech Republic; 9 Department of Pathology and Molecular Medicine, 2nd Faculty of Medicine and Motol University Hospital, Prague, Czech Republic; 10 Department of Neurology and Epileptology, The Children’s Memorial Health Institute, and Department of Child Neurology, Warsaw Medical University, Warsaw, Poland; 11 Pediatric Neurology Unit - UZ Brussel, Brussels, Belgium; 12 Department of Development and Regeneration-Section Pediatric Neurology, University Hospitals KU Leuven, Leuven, Belgium; 13 Systems Medicine Department, Child Neurology and Psychiatry Unit, Tor Vergata University Hospital of Rome, Rome, Italy; 14 Department of Medicine, Brigham and Women’s Hospital, Boston, MA USA; 15 Department of Translational Neuroscience, Brain Center Rudolf Magnus, University Medical Center Utrecht, Utrecht, The Netherlands; 16 Swammerdam Institute for Life Sciences, Center for Neuroscience, University of Amsterdam, Amsterdam, The Netherlands; 17 Department of Neurology, University of Maryland School of Medicine, Baltimore, MD USA; 18 Center for Experimental & Molecular Medicine, and Department of Clinical Epidemiology, Biostatistics and Bioinformatics, Academic Medical Center University of Amsterdam, Amsterdam, The Netherlands; 19 Stichting Epilepsie Instellingen Nederland (SEIN), Heemstede, The Netherlands

Correction to: *Scientific Reports* 10.1038/s41598-017-06145-8, published online 14 August 2017

This Article contains an error in the Acknowledgements section.

“This work was supported by European Union’s Seventh Framework Programme (FP7/2007-2013) under the grant agreement no. 602391 (project acronym EPISTOP; A.I., J.v.S., A.M., E.A., F.I., K.K., S.K., A.J., L.L., P.C., D.K.); Epilepsiefonds (WAR 15-05; RJP); European Union’s Horizon 2020 research and innovation programme under the Marie Sklodowska-Curie grant agreement No 642881 (ECMED; A.E., J.G.). None of the authors has any conflict of interest to disclose. We confirm that we have read the Journal’s position on issues involved in ethical publication and affirm that this report is consistent with those guidelines.”

should read:

“This work was supported by European Union’s Seventh Framework Programme (FP7/2007-2013) under the Grant Agreement no. 602391 (project acronym EPISTOP; A.I., J.v.S., A.M., E.A., F.I., K.K., S.J., A.J., L.L., P.C., D.K.) and partially supported by the Polish Ministerial funds for science (years 2013–2019) for the implementation of international co-financed project (K.K, S.J.); Epilepsiefonds (WAR 15-05; RJP); European Union’s Horizon 2020 research and innovation programme under the Marie Sklodowska-Curie grant agreement No 642881 (ECMED; A.E., J.G.). None of the authors has any conflict of interest to disclose. We confirm that we have read the Journal’s position on issues involved in ethical publication and affirm that this report is consistent with those guidelines.”

