# Supplementary material for: Coding and small non-coding transcriptional landscape of tuberous sclerosis complex cortical tubers: implications for pathophysiology and treatment
Source: Sci Rep. 2017 Aug 14;7:8089. doi: 10.1038/s41598-017-06145-8 (PMC5556011; doi:10.1038/s41598-017-06145-8)
Supplement: Supplementary file 1 — Supplementary PDF File [file 41598_2017_6145_MOESM1_ESM.pdf]

Supplementary Information:

Coding and small non-coding transcriptional landscape of tuberous sclerosis complex cortical tubers: implications for pathophysiology and treatment

James D. Mills[1a,†], Anand M. Iyer[1a,†], Jackelien van Scheppingen[1a], Anika Bongaarts[1a], Jasper J. Anink[1a], Bart Janssen[2], Till S. Zimmer[1a], Wim G. Spliet[3a], Peter. C. van Rijen[3b,d], Floor E. Jansen[3,d], Martha Feucht[4a], Johannes A. Hainfellner[4b], Pavel Krsek[5a], Josef Zamecnik[5b], Katarzyna Kotulska[6], Sergiusz Jozwiak[6], Anna Jansen[7], Lieven Lagae[8], Paolo Curatolo[9], David J. Kwiatkowski[10], R. Jeroen Pasterkamp[3d] , Ketharini Senthilkumar[3d], Lars von Oerthel[11], Marco F. Hoekman[11], Jan A. Gorter[11] Peter B. Crino[12], Angelika. Mühlebner[1a], Brendon P. Scicluna[1b,c,*], Eleonora Aronica[1a,11,13,*].

[1]Department of (Neuro)Pathology[a], Center for Experimental & Molecular Medicine[b] and Department of Clinical Epidemiology, Biostatistics and Bioinformatics, Academic Medical Center, University of Amsterdam, Amsterdam, the Netherlands.

[2]GenomeScan BV, Leiden, the Netherlands.

[3]Departments of Pathology[a] and Neurosurgery[b], Pediatric Neurology[c] and Translational Neuroscience[d], Brain Center Rudolf Magnus, University Medical Center Utrecht, The Netherlands

[4]Department of Pediatrics[a] and Institute of Neurology[b], Medical University Vienna, Austria

[5]Department of Pediatric Neurology[a] and Department of Pathology and Molecular Medicine[b], Charles University, Second Medical School, Motol University Hospital, Prague, Czech Republic

[6]Department of Neurology and Epileptology, The Children's Memorial Health Institute, and Department of Child Neurology, Warsaw Medical University, Warsaw, Poland

[7]Pediatric Neurology Unit - UZ Brussel, Brussels, Belgium

[8]Department of Development and Regeneration-Section Pediatric Neurology, University Hospitals KU Leuven, Leuven, Belgium

[9]Systems Medicine Department, Child Neurology and Psychiatry Unit, Tor Vergata University Hospital of Rome, Italy

[10]Department of Medicine, Brigham and Women's Hospital, Boston, Massachusetts, USA

[11]Swammerdam Institute for Life Sciences, Center for Neuroscience, University of Amsterdam, The Netherlands

[12]Department of Neurology, University of Maryland School of Medicine, Baltimore, MD, USA

[13]Stichting Epilepsie Instellingen Nederland (SEIN), The Netherlands

† ***Authors contributed equally to the present work***

* ***These authors share the senior authorship***

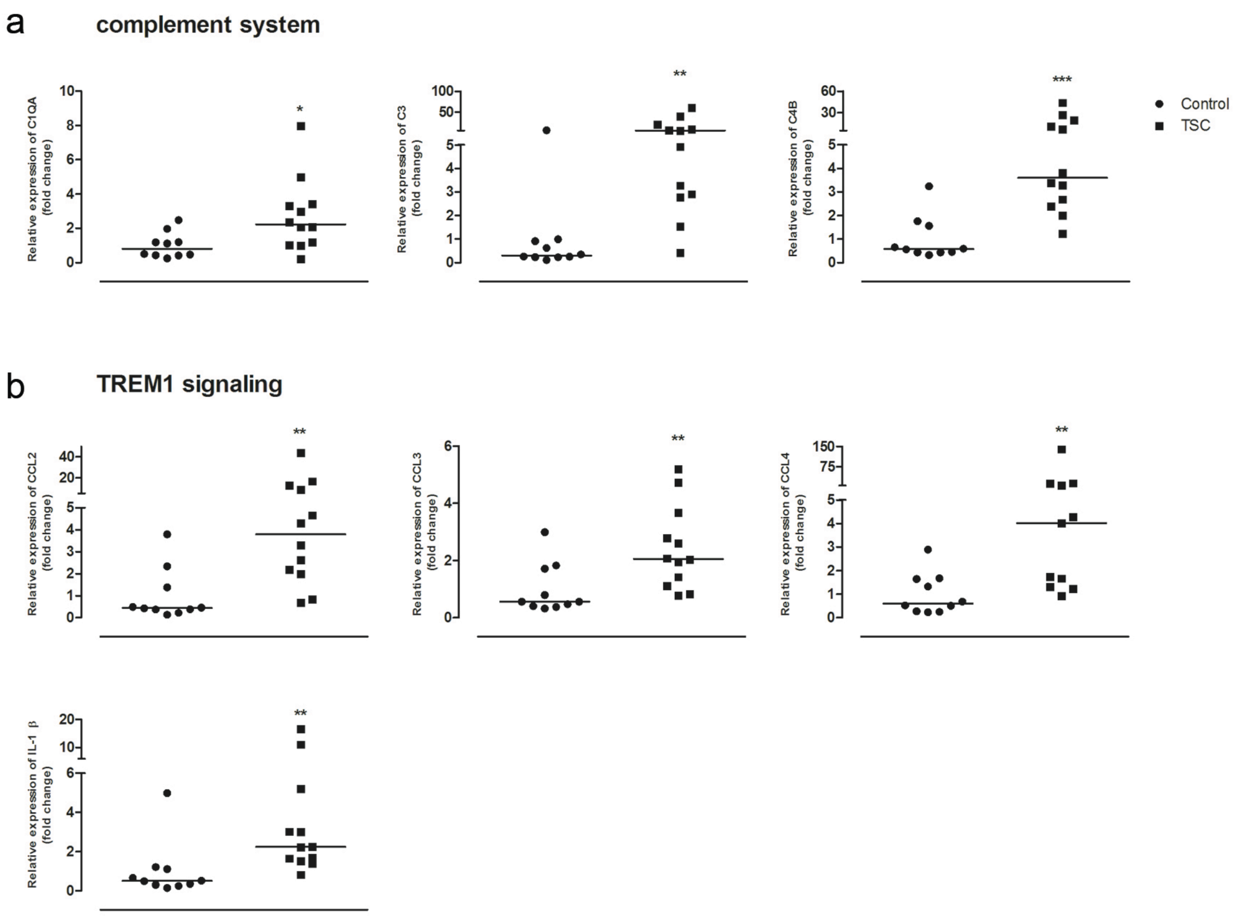

Supplementary Figure 1: Validation of selected genes of the complement system and TREM1 signaling in TSC cortical tubers as compared to control post-mortem cortex using RT-qPCR (a) *C1QA, C3* and *C4B* show significantly increased expression in TSC cortical tubers compared to control post-mortem cortex (b) *CCL2*, *CCL3*, *CCL4* and *IL1β* show significantly increased expression in TSC cortical tubers compared to control post-mortem cortex. Mann-Whitney U-test; *p<0.05, **p<0.01, ***p<0.001

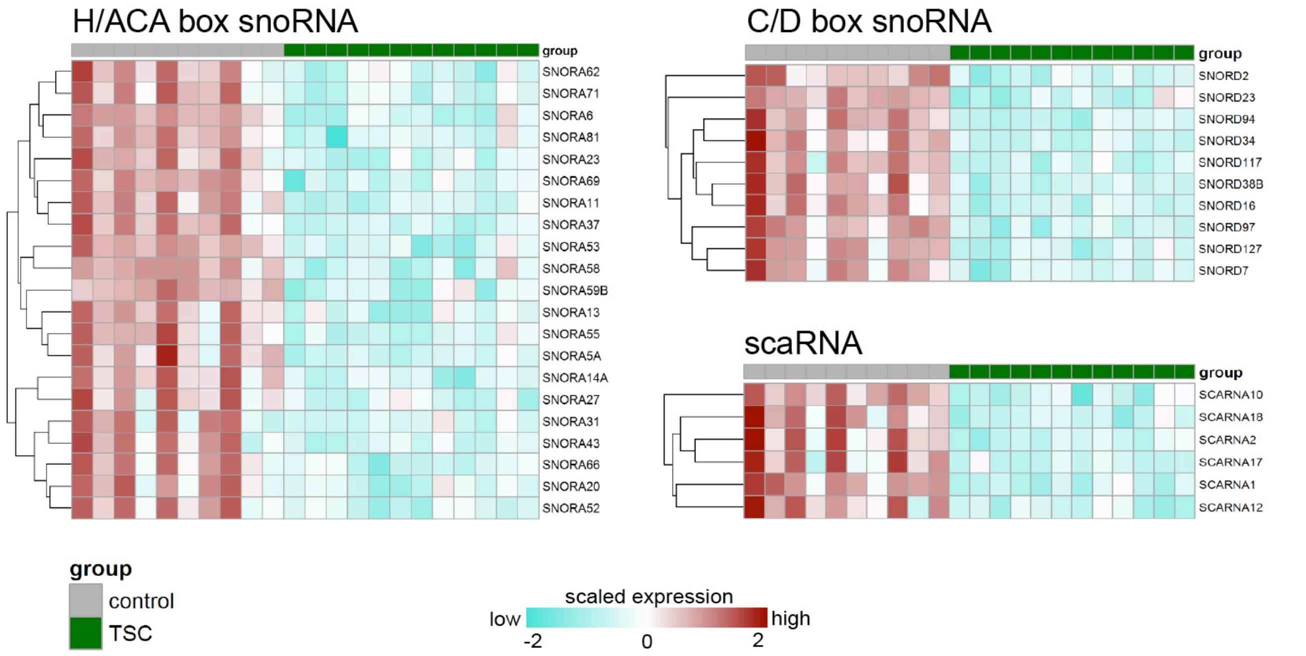

Supplementary Figure 2: Heat maps showing the expression of small non-coding RNAs other than miRNA in TSC cortical tubers compared to control post-mortem cortex. H/ACA box snoRNA, C/D box snoRNA and scaRNA showed significantly lower read counts in TSC cortical tubers compared to control post-mortem cortex.

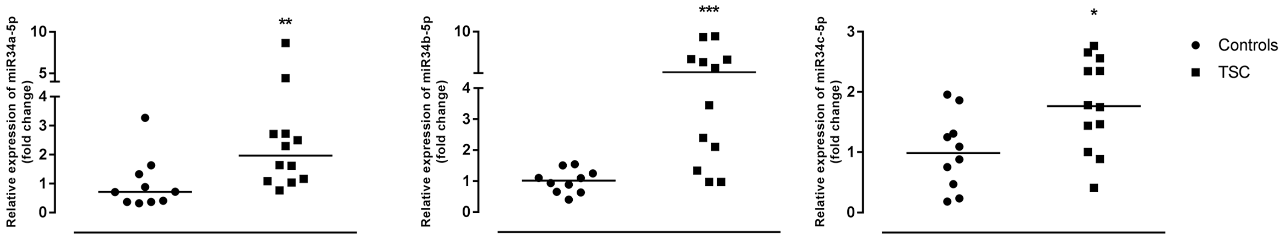

Supplementary Figure 3 Validation of selected differentially expressed miRNAs in TSC cortical tubers as compared to control post-mortem cortex using Taqman PCR Significantly increased expression of miR-34a-5p, miR-34b-5p and, miR-34c-5p was observed in TSC cortical tubers compared to control post-mortem cortex. Mann-Whitney U test; *p<0.05, **p<0.01, ***p<0.001.

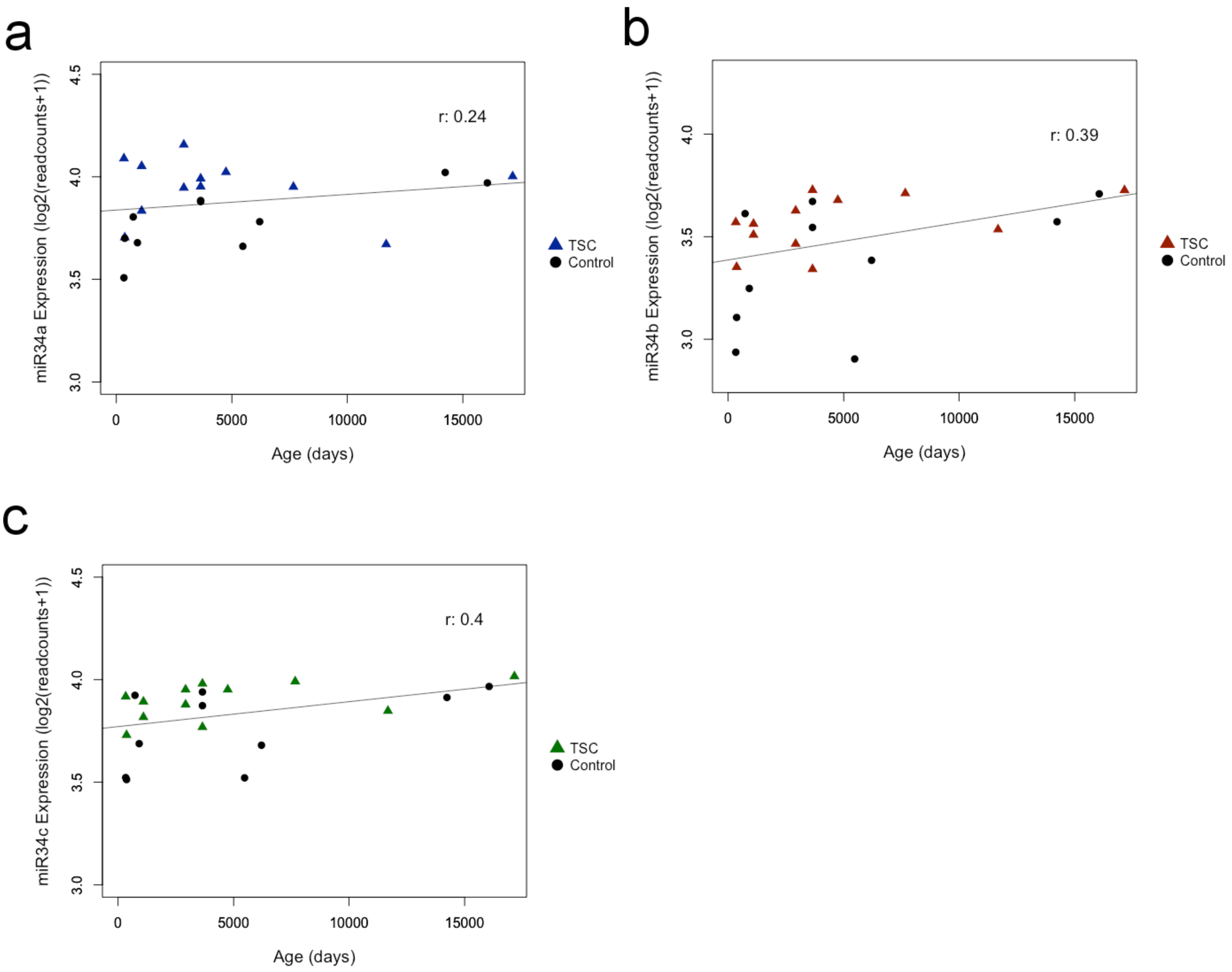

Supplementary Figure 4: Age independent expression of the miR-34 family. All members of the miR-34 family were tested for age dependent expression. All three miRNAs showed minimal correlation with age (Pearson's correlation coefficient). **a.** miR-34a (r=0.24, p-value>0.25) **b.** miR-34b (r=0.39, p-value>0.06) **c.** miR-34c (r=0.4, p-vlaue>0.06).

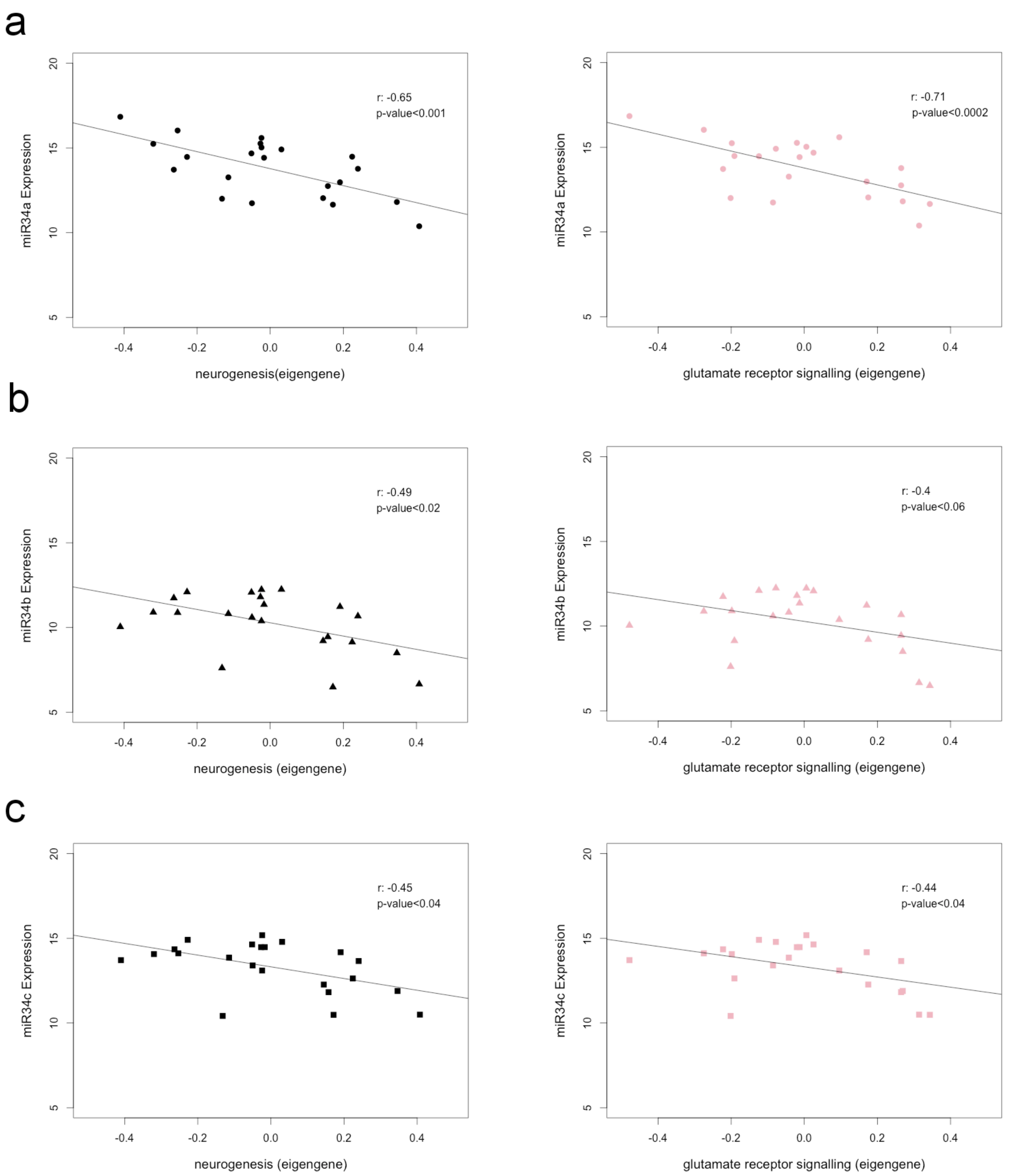

Supplementary figure 5: Correlation of each member of the miR-34 family with the modules neurogenesis and glutamate receptor signaling. All correlations are Pearson correlations. **a.** miR-34a: neurogenesis and glutamate receptor signaling. **b.** miR-34b:

neurogenesis and glutamate receptor signaling. **b.** miR-34c: neurogenesis and glutamate receptor signaling.

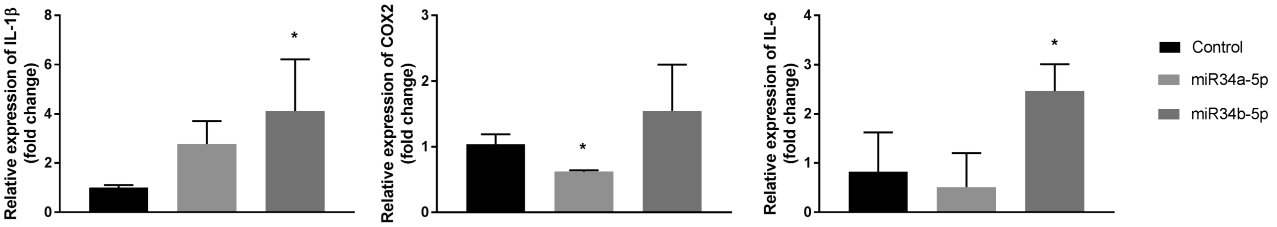

Supplementary figure 6: Expression levels of *IL1B, COX2* and *IL6* after transfection with miR-34a-5p and miR-34b-5p mimic. After transfection with the miR-34b-5p mimic there was a statistically significant increase in the expression of *IL1B* (~4-Fold, p-value<0.03) and *IL6* (~2.3-fold, p-value<0.03). There was also an increase in the expression of *COX2* however this was not statistically significant. Transfection with the miR-34a-5p mimic, resulted in no significant changes in the expression of *IL1B* and *IL6*, with a statistically significant decrease in *COX2* (~1.5-fold, p-value<0.03). Mann-Whitney U test.

Supplementary Table 1: Differentially expressed cell specific genes

Cell Type	Direction	Genes
Microglia	Up	*AIF1*<br>*C1QA*<br>*C1QB*<br>*C1QC*<br>*CCL2*<br>*CH25H*<br>*CX3CR1*<br>*HCK*<br>*IL1A*<br>*IRF5*<br>*ITGB2*<br>*LILRA4*<br>*LPAR5*<br>*MS4A6A*<br>*PIK3R5*<br>*PLVAP*<br>*RGS10*<br>*RNASE6*<br>*S100A11*<br>*SERPINA1*<br>*TNFRSF12A*<br>*TREM2*<br>*TYROBP*
Microglia	Down	*RP11-108M9.3*
Astrocytes	Up	*AEBP1*<br>*APLNR*<br>*C1QTNF5*<br>*C4B*<br>*COPZ2*<br>*GFAP*<br>*ITGB4*<br>*PLEKHA4*
Astrocytes	Down	*GJB2*<br>*PRSS35*
Neurons	Up	*AC144835.1*<br>*ARHGAP36*<br>*ARRDC4*<br>*C21orf88*<br>*PPP1R17*
Neurons	Down	*C11orf95*<br>*C1orf95*<br>*CRH*<br>*IGFBP3*<br>*SLC22A8*<br>*SSTR1*
Oligodendrocytes	Up	*CHI3L2*<br>*FAM46B*<br>*PIFO*
Oligodendrocytes	Down	*LPPR3*

Supplementary Table 2: Co-expression modules

module	# genes	GO biological process	adjusted P
turquoise	1391	negative regulation of dendrite development	0.018
blue	773	oligodendrocyte differentiation	0.00025
brown	712	Angiogenesis	0.0081
yellow	681	not determined	-
green	652	innate immune response	1.21x10-22
red	646	synaptic signaling	9.1x10-8
black	628	Neurogenesis	8.1x10-5
pink	608	glutamate receptor signaling	0.0039
magenta	477	extracellular matrix organization	0.002
purple	435	nucleobase-containing compound metabolic process	0.042
greenyellow	372	not determined	-
tan	360	not determined	-
salmon	355	not determined	-
cyan	315	not determined	-
midnightblue	284	not determined	-
lightcyan	268	not determined	-
grey60	231	not determined	-
lightgreen	183	nervous system development	0.026
lightyellow	172	type I interferon signaling pathway	1.5x10-8
royalblue	161	not determined	-
darkred	148	not determined	-
unclassified	148	-	-

Supplementary Table 3: Correlation of miRNAs with modules enriched for specific gene ontology terms. The top value in each cell is the Pearson's correlation coefficient, the lower value in brackets is the associated p-value.

	Innate immune response	Oligodendrocyte differentiation	Type I interferon signaling pathway	Extracellular matrix organisation	Nucleobase-contating compound metablic process	Synaptic signaling	Neurogenesis	Angiogenesis	Negative regulation of dendrite development	Nervous system development	Glutamate receptor signaling
MIR100	0.54<br>(0.010)	0.34<br>(0.120)	0.31<br>(0.162)	0.65<br>(0.001)	-0.19<br>(0.388)	-0.13<br>(0.552)	-0.38<br>(0.078)	-0.24<br>(0.291)	-0.30<br>(0.176)	-0.52<br>(0.013)	-0.47<br>(0.029)
MIR1262	0.57<br>(0.006)	-0.06<br>(0.807)	0.42<br>(0.053)	0.65<br>(0.001)	-0.25<br>(0.268)	-0.11<br>(0.641)	-0.23<br>(0.301)	-0.03<br>(0.894)	-0.24<br>(0.287)	-0.56<br>(0.006)	-0.40<br>(0.064)
MIR141	0.65<br>(0.001)	0.11<br>(0.623)	0.48<br>(0.025)	0.66<br>(0.001)	-0.28<br>(0.200)	-0.12<br>(0.585)	-0.43<br>(0.047)	-0.16<br>(0.475)	-0.25<br>(0.256)	-0.58<br>(0.005)	-0.51<br>(0.014)
MIR142	0.77<br>(0.000)	0.18<br>(0.419)	0.57<br>(0.006)	0.63<br>(0.002)	-0.22<br>(0.327)	0.05<br>(0.827)	-0.42<br>(0.050)	-0.22<br>(0.319)	-0.28<br>(0.204)	-0.57<br>(0.006)	-0.41<br>(0.057)
MIR151B	0.37<br>(0.094)	0.66<br>(0.001)	0.39<br>(0.074)	0.45<br>(0.037)	0.06<br>(0.776)	0.00<br>(0.989)	-0.43<br>(0.049)	-0.40<br>(0.062)	-0.32<br>(0.145)	-0.32<br>(0.153)	-0.37<br>(0.088)
MIR152	0.66<br>(0.001)	0.17<br>(0.462)	0.60<br>(0.003)	0.81<br>(0.000)	-0.10<br>(0.671)	-0.21<br>(0.356)	-0.47<br>(0.029)	-0.29<br>(0.194)	-0.50<br>(0.019)	-0.68<br>(0.000)	-0.63<br>(0.002)
MIR182	0.60<br>(0.003)	0.00<br>(0.989)	0.34<br>(0.122)	0.37<br>(0.089)	-0.21<br>(0.354)	0.15<br>(0.495)	-0.15<br>(0.504)	0.02<br>(0.935)	-0.05<br>(0.830)	-0.32<br>(0.146)	-0.12<br>(0.585)
MIR193A	0.69<br>(0.000)	0.13<br>(0.563)	0.52<br>(0.012)	0.83<br>(0.000)	-0.19<br>(0.403)	-0.19<br>(0.393)	-0.49<br>(0.022)	-0.25<br>(0.258)	-0.45<br>(0.036)	-0.73<br>(0.000)	-0.65<br>(0.001)
MIR193B	0.56<br>(0.007)	0.29<br>(0.186)	0.50<br>(0.017)	0.66<br>(0.001)	-0.12<br>(0.606)	-0.30<br>(0.168)	-0.48<br>(0.025)	-0.36<br>(0.095)	-0.46<br>(0.032)	-0.62<br>(0.002)	-0.59<br>(0.004)
MIR200A	0.58<br>(0.005)	0.48<br>(0.022)	0.55<br>(0.008)	0.66<br>(0.001)	0.00<br>(0.983)	-0.06<br>(0.790)	-0.54<br>(0.010)	-0.43<br>(0.047)	-0.45<br>(0.035)	-0.56<br>(0.007)	-0.55<br>(0.008)
MIR200B	0.74<br>(0.000)	0.24<br>(0.292)	0.51<br>(0.015)	0.72<br>(0.000)	-0.19<br>(0.405)	-0.08<br>(0.737)	-0.42<br>(0.053)	-0.16<br>(0.481)	-0.30<br>(0.176)	-0.62<br>(0.002)	-0.51<br>(0.016)
MIR200C	0.47<br>(0.026)	0.16<br>(0.490)	0.43<br>(0.045)	0.58<br>(0.005)	-0.13<br>(0.572)	-0.14<br>(0.525)	-0.44<br>(0.039)	-0.28<br>(0.210)	-0.33<br>(0.129)	-0.46<br>(0.030)	-0.48<br>(0.024)
MIR208B	0.46<br>(0.032)	0.05<br>(0.841)	0.19<br>(0.386)	0.59<br>(0.004)	-0.20<br>(0.372)	-0.21<br>(0.351)	-0.20<br>(0.368)	-0.15<br>(0.509)	-0.32<br>(0.141)	-0.53<br>(0.012)	-0.40<br>(0.063)
MIR21	0.76<br>(0.000)	0.09<br>(0.677)	0.62<br>(0.002)	0.74<br>(0.000)	-0.32<br>(0.150)	-0.31<br>(0.156)	-0.61<br>(0.003)	-0.36<br>(0.097)	-0.46<br>(0.032)	-0.77<br>(0.000)	-0.67<br>(0.001)
MIR2114	0.38<br>(0.080)	0.20<br>(0.361)	0.48<br>(0.023)	0.69<br>(0.000)	0.02<br>(0.921)	-0.25<br>(0.262)	-0.39<br>(0.072)	-0.31<br>(0.166)	-0.47<br>(0.027)	-0.52<br>(0.014)	-0.56<br>(0.006)
MIR215	0.52<br>(0.012)	0.03<br>(0.886)	0.33<br>(0.131)	0.57<br>(0.005)	-0.12<br>(0.593)	-0.14<br>(0.531)	-0.15<br>(0.515)	0.00<br>(0.998)	-0.24<br>(0.284)	-0.45<br>(0.034)	-0.34<br>(0.122)
MIR219A1	0.43<br>(0.048)	0.80<br>(0.000)	0.51<br>(0.016)	0.47<br>(0.027)	0.23<br>(0.306)	0.14<br>(0.537)	-0.45<br>(0.036)	-0.42<br>(0.054)	-0.34<br>(0.120)	-0.27<br>(0.226)	-0.34<br>(0.119)
MIR219B	0.37<br>(0.087)	0.81<br>(0.000)	0.50<br>(0.018)	0.40<br>(0.062)	0.23<br>(0.305)	0.07<br>(0.741)	-0.49<br>(0.021)	-0.50<br>(0.017)	-0.38<br>(0.081)	-0.26<br>(0.238)	-0.35<br>(0.106)
MIR223	0.72<br>(0.000)	0.32<br>(0.148)	0.60<br>(0.003)	0.46<br>(0.031)	-0.25<br>(0.256)	-0.02<br>(0.938)	-0.57<br>(0.005)	-0.38<br>(0.082)	-0.28<br>(0.212)	-0.52<br>(0.012)	-0.44<br>(0.041)
MIR2276	0.71<br>(0.000)	0.27<br>(0.216)	0.66<br>(0.001)	0.67<br>(0.001)	-0.06<br>(0.782)	-0.04<br>(0.877)	-0.55<br>(0.007)	-0.29<br>(0.192)	-0.37<br>(0.093)	-0.62<br>(0.002)	-0.57<br>(0.006)
MIR25	0.57<br>(0.006)	0.32<br>(0.153)	0.48<br>(0.024)	0.56<br>(0.006)	-0.14<br>(0.526)	-0.06<br>(0.788)	-0.41<br>(0.059)	-0.28<br>(0.199)	-0.30<br>(0.178)	-0.47<br>(0.027)	-0.41<br>(0.057)
MIR302A	0.40<br>(0.063)	0.36<br>(0.096)	0.43<br>(0.048)	0.64<br>(0.001)	0.07<br>(0.749)	-0.15<br>(0.501)	-0.36<br>(0.100)	-0.29<br>(0.188)	-0.45<br>(0.036)	-0.46<br>(0.030)	-0.50<br>(0.019)
MIR3129	0.47<br>(0.026)	0.20<br>(0.372)	0.51<br>(0.014)	0.44<br>(0.042)	-0.27<br>(0.216)	-0.07<br>(0.749)	-0.55<br>(0.008)	-0.31<br>(0.157)	-0.18<br>(0.416)	-0.40<br>(0.069)	-0.43<br>(0.044)
MIR33B	0.34<br>(0.119)	0.70<br>(0.000)	0.42<br>(0.049)	0.46<br>(0.030)	0.17<br>(0.441)	-0.04<br>(0.857)	-0.44<br>(0.038)	-0.45<br>(0.034)	-0.40<br>(0.063)	-0.32<br>(0.140)	-0.42<br>(0.053)
MIR34A	0.74	0.22	0.70	0.71	-0.17	-0.18	-0.65	-0.41	-0.45	-0.71	-0.65

	(0.000)	(0.327)	(0.000)	(0.000)	(0.436)	(0.418)	(0.001)	(0.060)	(0.034)	(0.000)	(0.001)
MIR34B	0.38 (0.078)	0.75 (0.000)	0.46 (0.032)	0.45 (0.038)	0.13 (0.577)	0.01 (0.953)	-0.49 (0.019)	-0.47 (0.028)	-0.37 (0.093)	-0.32 (0.151)	-0.40 (0.062)
MIR34C	0.46 (0.033)	0.72 (0.000)	0.50 (0.018)	0.51 (0.016)	0.10 (0.650)	-0.03 (0.893)	-0.51 (0.016)	-0.45 (0.038)	-0.39 (0.076)	-0.37 (0.087)	-0.44 (0.039)
MIR3617	0.40 (0.065)	0.41 (0.056)	0.37 (0.087)	0.61 (0.003)	0.22 (0.319)	0.12 (0.599)	-0.20 (0.366)	-0.20 (0.378)	-0.39 (0.075)	-0.37 (0.088)	-0.36 (0.098)
MIR362	0.69 (0.000)	0.01 (0.952)	0.49 (0.022)	0.58 (0.004)	-0.32 (0.151)	-0.09 (0.698)	-0.45 (0.037)	-0.25 (0.257)	-0.29 (0.187)	-0.61 (0.003)	-0.46 (0.032)
MIR3622B	0.60 (0.003)	0.14 (0.530)	0.40 (0.069)	0.80 (0.000)	-0.06 (0.776)	-0.10 (0.666)	-0.29 (0.185)	-0.12 (0.597)	-0.38 (0.081)	-0.61 (0.002)	-0.50 (0.017)
MIR3917	0.34 (0.121)	-0.08 (0.721)	0.05 (0.835)	0.19 (0.392)	-0.47 (0.026)	-0.07 (0.766)	-0.11 (0.639)	-0.02 (0.917)	0.05 (0.839)	-0.28 (0.201)	-0.09 (0.700)
MIR429	0.66 (0.001)	0.34 (0.127)	0.52 (0.013)	0.62 (0.002)	-0.15 (0.494)	-0.03 (0.898)	-0.50 (0.018)	-0.29 (0.195)	-0.32 (0.146)	-0.55 (0.008)	-0.50 (0.018)
MIR455	0.66 (0.001)	-0.04 (0.846)	0.51 (0.016)	0.65 (0.001)	-0.30 (0.168)	-0.14 (0.532)	-0.43 (0.045)	-0.17 (0.438)	-0.30 (0.176)	-0.65 (0.001)	-0.53 (0.011)
MIR4671	0.42 (0.052)	0.38 (0.079)	0.11 (0.625)	0.39 (0.077)	-0.16 (0.468)	0.10 (0.672)	-0.13 (0.563)	-0.03 (0.903)	-0.05 (0.820)	-0.28 (0.206)	-0.16 (0.491)
MIR4677	0.66 (0.001)	0.28 (0.209)	0.44 (0.040)	0.72 (0.000)	-0.15 (0.517)	-0.17 (0.438)	-0.42 (0.051)	-0.29 (0.194)	-0.42 (0.050)	-0.63 (0.002	-0.55 (0.008)
MIR4796	0.46 (0.029)	0.15 (0.502)	0.41 (0.060)	0.43 (0.045)	-0.31 (0.157)	-0.19 (0.387)	-0.45 (0.036)	-0.24 (0.272)	-0.19 (0.387)	-0.43 (0.046)	-0.41 (0.059)
MIR501	0.57 (0.006)	-0.13 (0.568)	0.19 (0.386)	0.68 (0.001)	-0.31 (0.154)	-0.22 (0.319)	-0.21 (0.357)	-0.05 (0.834)	-0.29 (0.198)	-0.61 (0.003)	-0.42 (0.052)
MIR502	0.72 (0.000)	0.18 (0.411)	0.65 (0.001)	0.75 (0.000)	-0.19 (0.386)	-0.20 (0.369)	-0.61 (0.003)	-0.37 (0.086)	-0.46 (0.030)	-0.71 (0.000)	-0.65 (0.001)
MIR548K	0.72 (0.000)	-0.11 (0.639)	0.37 (0.086)	0.71 (0.000)	-0.42 (0.054)	-0.29 (0.187)	-0.35 (0.106)	-0.11 (0.616)	-0.31 (0.160)	-0.73 (0.000)	-0.53 (0.010)
MIR548N	0.66 (0.001)	-0.33 (0.128)	0.28 (0.208)	0.52 (0.014)	-0.46 (0.029)	-0.30 (0.169)	-0.20 (0.380)	0.07 (0.744)	-0.16 (0.485)	-0.63 (0.002)	-0.40 (0.066)
MIR5695	0.43 (0.044)	0.07 (0.756)	0.03 (0.880)	0.48 (0.024)	-0.12 (0.592)	0.15 (0.508)	0.13 (0.559)	0.31 (0.165)	0.07 (0.759)	-0.25 (0.259)	-0.06 (0.781)
MIR574	0.47 (0.029)	0.59 (0.004)	0.52 (0.012)	0.53 (0.010)	0.05 (0.834)	-0.02 (0.942)	-0.53 (0.011)	-0.45 (0.036)	-0.39 (0.070)	-0.42 (0.052)	-0.48 (0.024)
MIR577	0.37 (0.094)	0.77 (0.000)	0.47 (0.029)	0.42 (0.051)	0.21 (0.355)	-0.01 (0.964)	-0.49 (0.020)	-0.53 (0.011)	-0.43 (0.046)	-0.31 (0.156)	-0.40 (0.066)
MIR584	0.33 (0.128)	0.77 (0.000)	0.44 (0.042)	0.41 (0.059)	0.20 (0.373)	0.00 (0.991)	-0.48 (0.025)	-0.50 (0.017)	-0.40 (0.067)	-0.29 (0.194)	-0.38 (0.080)
MIR616	0.68 (0.001)	0.28 (0.202)	0.71 (0.000)	0.60 (0.003)	-0.13 (0.562)	-0.18 (0.414)	-0.66 (0.001)	-0.40 (0.068)	-0.40 (0.063)	-0.60 (0.003)	-0.61 (0.002)
MIR6504	0.54 (0.009)	0.09 (0.698)	0.38 (0.077)	0.49 (0.020)	-0.04 (0.870)	-0.07 (0.741)	-0.40 (0.067)	-0.32 (0.146)	-0.42 (0.051)	-0.51 (0.015)	-0.42 (0.053)
MIR6513	0.59 (0.004)	0.25 (0.261)	0.64 (0.001)	0.69 (0.000)	0.05 (0.840)	0.01 (0.970)	-0.48 (0.025)	-0.31 (0.156)	-0.44 (0.042	-0.53 (0.012)	-0.52 (0.013)
MIR652	0.52 (0.014)	0.10 (0.660)	0.44 (0.039)	0.65 (0.001)	-0.05 (0.820)	-0.24 (0.273)	-0.31 (0.159)	-0.28 (0.213)	-0.48 (0.023)	-0.60 (0.003)	-0.52 (0.014)

Supplementary Table 4: Predicted targets of the miR-34 family amongst the modules neurogenesis and glutamate receptor signaling.

miR-34a hits				
**Gene ID**	**Gene Name**	**Module**	**FC**	**DEG**
ARHGAP19	Rho GTPase activating protein 19	neurogenesis	-0.66	no
SATB2-AS1	SATB2 antisense RNA 1	neurogenesis	-0.87	no
WNT10B	Wnt family member 10B	neurogenesis	-0.50	no
DNAJC5G	DnaJ heat shock protein family (Hsp40) member C5 gamma	neurogenesis	-0.99	no
SNAP23	synaptosome associated protein 23	neurogenesis	0.25	no
ARHGAP4	Rho GTPase activating protein 4	neurogenesis	-0.27	no
ARHGAP33	Rho GTPase activating protein 33	neurogenesis	-1.08	yes
RPS24P7	ribosomal protein S24 pseudogene 7	neurogenesis	-0.80	no
ARL4D	ADP ribosylation factor like GTPase 4D	neurogenesis	-0.67	no
SERPINB1	serpin family B member 1	neurogenesis	0.86	no
TGFBR2	transforming growth factor beta receptor 2	neurogenesis	0.28	no
PTMS	parathymosin	neurogenesis	-0.51	no
UBE2S	ubiquitin conjugating enzyme E2 S	neurogenesis	-0.36	no
ARL16	ADP ribosylation factor like GTPase 16	neurogenesis	-0.09	no
TPD52L1	tumor protein D52-like 1	neurogenesis	1.45	no
ZFR2	zinc finger RNA binding protein 2	neurogenesis	-0.65	no
ADARB1	adenosine deaminase, RNA specific B1	neurogenesis	-0.37	no
BCL2	BCL2, apoptosis regulator	neurogenesis	0.30	no
NAMPTL	Nicotinamide Phosphoribosyltransferase Pseudogene 1	neurogenesis	-0.36	no
GRM7	glutamate metabotropic receptor 7	neurogenesis	-0.63	no
ARL8A	ADP ribosylation factor like GTPase 8A	neurogenesis	-0.22	no
UST	uronyl 2-sulfotransferase	neurogenesis	-0.21	no
TGM2	transglutaminase 2	neurogenesis	-1.05	no
VCL	vinculin	neurogenesis	-0.11	no
ARC	activity regulated cytoskeleton associated protein	neurogenesis	-0.31	no
APOL2	apolipoprotein L2	neurogenesis	0.11	no
H3F3AP4	H3 histone, family 3A, pseudogene 4	neurogenesis	-1.28	no
ITGA6	integrin subunit alpha 6	glutamate receptor signaling	0.74	no
ARPP21	cAMP regulated phosphoprotein 21	glutamate receptor signaling	-0.81	no
RTN4RL1	reticulon 4 receptor like 1	glutamate receptor signaling	-0.47	no
CADPS	calcium dependent secretion activator	glutamate receptor signaling	-0.30	no
DOCK3	dedicator of cytokinesis 3	glutamate receptor signaling	-0.66	no
ARHGAP44	Rho GTPase activating protein 44	glutamate receptor signaling	-0.49	no
SATB2	SATB homeobox 2	glutamate receptor signaling	-1.13	no
ARNTL2	aryl hydrocarbon receptor nuclear translocator like 2	glutamate receptor signaling	-0.73	no
NEUROD2	neuronal differentiation 2	glutamate receptor signaling	-0.51	no
MAP3K9	mitogen-activated protein kinase kinase kinase 9	glutamate receptor signaling	-0.77	no
ARHGEF7	Rho guanine nucleotide exchange factor 7	glutamate receptor signaling	-0.48	no
DDA1	DET1 and DDB1 associated 1	glutamate receptor signaling	-0.26	no
BCL2L12	BCL2 like 12	glutamate receptor signaling	0.67	no
ARHGEF11	Rho guanine nucleotide exchange factor 11	glutamate receptor signaling	-0.47	no

ARSD-AS1	ARSD antisense RNA 1	glutamate receptor signaling	0.35	no
CPEB3	cytoplasmic polyadenylation element binding protein 3	glutamate receptor signaling	-0.31	no
ARHGAP10	Rho GTPase activating protein 10	glutamate receptor signaling	-0.82	no
CALD1	caldesmon 1	glutamate receptor signaling	-0.21	no
ARMCX6	armadillo repeat containing, X-linked 6	glutamate receptor signaling	0.66	no
MCM5	minichromosome maintenance complex component 5	glutamate receptor signaling	1.03	no
MBD6	methyl-CpG binding domain protein 6	glutamate receptor signaling	0.09	no
CD248	CD248 molecule	glutamate receptor signaling	0.24	no
HIST1H4J	histone cluster 1 H4 family member j	glutamate receptor signaling	0.22	no
MYBPC2	myosin binding protein C, fast type	glutamate receptor signaling	-0.71	no
**miR-34b hits**				
**Gene ID**	**Gene Name**	**Module**	**FC**	**DEG**
BAIAP2-AS1	BAIAP2 antisense RNA 1 (head to head)	neurogenesis	-0.43	no
ASPHD2	aspartate beta-hydroxylase domain containing 2	neurogenesis	-0.54	no
ARHGAP19	Rho GTPase activating protein 19	neurogenesis	-0.66	no
TMEM198	transmembrane protein 198	neurogenesis	-0.28	no
CELF3	CUGBP Elav-like family member 3	neurogenesis	-1.29	no
GSG1	germ cell associated 1	neurogenesis	-1.95	no
RPS6KL1	ribosomal protein S6 kinase like 1	neurogenesis	-0.12	no
PVRL3	Nectin Cell Adhesion Molecule 3	neurogenesis	-1.01	no
MESP1	mesoderm posterior bHLH transcription factor 1	neurogenesis	-0.15	no
MYO16	myosin XVI	neurogenesis	-1.45	no
TGFBR2	transforming growth factor beta receptor 2	neurogenesis	0.28	no
SPP1	secreted phosphoprotein 1	neurogenesis	2.07	yes
CKMT2	creatine kinase, mitochondrial 2	neurogenesis	0.46	no
BCL2	BCL2, apoptosis regulator	neurogenesis	0.30	no
NR4A3	nuclear receptor subfamily 4 group A member 3	neurogenesis	-0.49	no
GYPE	glycophorin E (MNS blood group)	neurogenesis	-0.45	no
SIGLEC14	sialic acid binding Ig like lectin 14	neurogenesis	0.63	no
TRIM25	tripartite motif containing 25	neurogenesis	-0.01	no
NEDD4L	neural precursor cell expressed, developmentally down-regulated 4-like, E3 ubiquitin protein ligase	neurogenesis	0.01	no
TAGLN	transgelin	neurogenesis	-0.14	no
CALCRL	calcitonin receptor like receptor	neurogenesis	-0.35	no
ADD2	adducin 2	glutamate receptor signaling	-0.98	yes
KIAA1549L	KIAA1549 like	glutamate receptor signaling	-0.67	no
CNTN3	contactin 3	glutamate receptor signaling	-1.32	yes
CACNG8	calcium voltage-gated channel auxiliary subunit gamma 8	glutamate receptor signaling	-0.72	no
ACTL6A	actin like 6A	glutamate receptor signaling	0.46	no
RAP1GAP2	RAP1 GTPase activating protein 2	glutamate receptor signaling	-0.76	no
CCDC85A	coiled-coil domain containing 85A	glutamate receptor signaling	-0.64	no
MAP3K9	mitogen-activated protein kinase kinase kinase 9	glutamate receptor signaling	-0.77	no
PTPN4	protein tyrosine phosphatase, non-receptor type 4	glutamate receptor signaling	-0.44	no
DDA1	DET1 and DDB1 associated 1	glutamate receptor signaling	-0.26	no
KCNJ11	potassium voltage-gated channel subfamily J member 11	glutamate receptor signaling	-0.59	no
BCL2L12	BCL2 like 12	glutamate receptor signaling	0.67	no
FAM132B	Family With Sequence Similarity 132 Member A	glutamate receptor signaling	-0.94	no

ARHGAP10	Rho GTPase activating protein 10	glutamate receptor signaling	-0.82	no
PLCE1	phospholipase C epsilon 1	glutamate receptor signaling	0.80	no
RPL23AP53	ribosomal protein L23a pseudogene 53	glutamate receptor signaling	-0.29	no
MAPT-IT1	MAPT intronic transcript 1	glutamate receptor signaling	-0.46	no
TMEM255A	transmembrane protein 255A	glutamate receptor signaling	1.08	no
PLEKHG5,TNFRSF25	Pleckstrin Homology And RhoGEF Domain Containing G5, TNF Receptor Superfamily Member 25	glutamate receptor signaling	-0.53	no
BTG2	BTG anti-proliferation factor 2	glutamate receptor signaling	1.10	yes
IFIT3	interferon induced protein with tetratricopeptide repeats 3	glutamate receptor signaling	0.01	no
CRB2	crumbs 2, cell polarity complex component	glutamate receptor signaling	1.14	no
SLC25A13	solute carrier family 25 member 13	glutamate receptor signaling	0.77	no
RDH12	retinol dehydrogenase 12 (all-trans/9-cis/11-cis)	glutamate receptor signaling	-0.40	no
CRTAC1	cartilage acidic protein 1	glutamate receptor signaling	0.07	no
ABHD17AP6	abhydrolase domain containing 17A pseudogene 6	glutamate receptor signaling	-0.57	no
ABHD1	abhydrolase domain containing 1	glutamate receptor signaling	0.45	no
CREB3L1	cAMP responsive element binding protein 3 like 1	glutamate receptor signaling	-0.10	no
PVRL3-AS1,RP11-553A10.1	PVRL3 Antisense RNA 1	glutamate receptor signaling	-0.93	no
PDLIM7	PDZ and LIM domain 7	glutamate receptor signaling	0.24	no
CAV1	caveolin 1	glutamate receptor signaling	1.08	no
MYBPC2	myosin binding protein C, fast type	glutamate receptor signaling	-0.71	no
RBM3	RNA binding motif (RNP1, RRM) protein 3	glutamate receptor signaling	-0.02	no
**miR-34c hits**				
**Gene ID**	**Gene Name**	**Module**	**FC**	**DEG**
ARHGAP19	Rho GTPase activating protein 19	neurogenesis	-0.66	no
SATB2-AS1	SATB2 antisense RNA 1	neurogenesis	-0.87	no
TGFBR2	transforming growth factor beta receptor 2	neurogenesis	0.28	no
CDH10	cadherin 10	neurogenesis	-0.87	no
RELL1	RELT like 1	neurogenesis	-0.31	no
BCL2	BCL2, apoptosis regulator	neurogenesis	0.30	no
UST	uronyl 2-sulfotransferase	neurogenesis	-0.21	no
CDH1	cadherin 1	neurogenesis	0.58	no
SATB2	SATB homeobox 2	glutamate receptor signaling	-1.13	no
PER1	period circadian clock 1	glutamate receptor signaling	-0.22	no
NEUROD2	neuronal differentiation 2	glutamate receptor signaling	-0.51	no
DDA1	DET1 and DDB1 associated 1	glutamate receptor signaling	-0.26	no
BCL2L12	BCL2 like 12	glutamate receptor signaling	0.67	no
CPEB3	cytoplasmic polyadenylation element binding protein 3	glutamate receptor signaling	-0.31	no
ARHGAP10	Rho GTPase activating protein 10	glutamate receptor signaling	-0.82	no
MAPT-IT1	MAPT intronic transcript 1	glutamate receptor signaling	-0.46	no
MBD6	methyl-CpG binding domain protein 6	glutamate receptor signaling	0.09	no
CAV1	caveolin 1	glutamate receptor signaling	1.08	no
MYBPC2	myosin binding protein C, fast type	glutamate receptor signaling	-0.71	no

Supplementary Table 5: Primers used for RT-qPCR

IL-1β	gcatccagctacgaatctcc	gaaccagcatcttcctcagc	99
IL-6	ctcagccctgagaaaggaga	tttcagccatctttggaagg	101
COX2	gaatggggtgatgagcagtt	gccactcaagtgttgcacat	99
CCL2	ctgctcatagcagccacctt	gcactgagatcttcctattggtg	106
CCL3	cagaatcatgcaggtctccac	gcgtgtcagcagcaagtg	95
CCL4	cttcctcgcaactttgtggt	tcactgggatcagcacagac	110
C1QA	caccagacgggaagaaagg	taaggccttggatgcctgt	117
C3	cctgaagatagagggtgacca	ccaccacgtcccagatctta	116
C4B	gctggagagtcagaccaagc	tggaggtcagtaatgcccata	108
EF1A	atccacctttgggtcgcttt	ccgcaactgtctgtctcatatcac	51
C1orf43	gatttccctgggtttccagt	attcgactctccagggttca	94
SNRPD3	atacagagatggccgagtgg	taacatgggtgcgttcttca	103
